# Sporozoite Immunization of Human Volunteers under Mefloquine Prophylaxis Is Safe, Immunogenic and Protective: A Double-Blind Randomized Controlled Clinical Trial

**DOI:** 10.1371/journal.pone.0112910

**Published:** 2014-11-14

**Authors:** Else M. Bijker, Remko Schats, Joshua M. Obiero, Marije C. Behet, Geert-Jan van Gemert, Marga van de Vegte-Bolmer, Wouter Graumans, Lisette van Lieshout, Guido J. H. Bastiaens, Karina Teelen, Cornelus C. Hermsen, Anja Scholzen, Leo G. Visser, Robert W. Sauerwein

**Affiliations:** 1 Radboud university medical center, Department of Medical Microbiology, PO Box 9101, 6500 HB Nijmegen, The Netherlands; 2 Leiden University Medical Center, Department of Infectious Diseases, PO Box 9600, 2300 RC Leiden, The Netherlands; 3 Leiden University Medical Center, Department of Medical Microbiology, PO Box 9600, 2300 RC Leiden, The Netherlands; 4 Leiden University Medical Center, Department of Parasitology, PO Box 9600, 2300 RC Leiden, The Netherlands; Kenya Medical Research Institute - Wellcome Trust Research Programme, Kenya

## Abstract

Immunization of healthy volunteers with chloroquine ChemoProphylaxis and Sporozoites (CPS-CQ) efficiently and reproducibly induces dose-dependent and long-lasting protection against homologous *Plasmodium falciparum* challenge. Here, we studied whether chloroquine can be replaced by mefloquine, which is the only other licensed anti-malarial chemoprophylactic drug that does not affect pre-erythrocytic stages, exposure to which is considered essential for induction of protection by CPS immunization. In a double blind randomized controlled clinical trial, volunteers under either chloroquine prophylaxis (CPS-CQ, n = 5) or mefloquine prophylaxis (CPS-MQ, n = 10) received three sub-optimal CPS immunizations by bites from eight *P. falciparum* infected mosquitoes each, at monthly intervals. Four control volunteers received mefloquine prophylaxis and bites from uninfected mosquitoes. CPS-MQ immunization is safe and equally potent compared to CPS-CQ inducing protection in 7/10 (70%) versus 3/5 (60%) volunteers, respectively. Furthermore, specific antibody levels and cellular immune memory responses were comparable between both groups. We therefore conclude that mefloquine and chloroquine are equally effective in CPS-induced immune responses and protection.

**Trial Registration:**

ClinicalTrials.gov NCT01422954

## Introduction

Malaria remains one of the most important infectious diseases worldwide and still causes approximately 207 million cases and 627,000 deaths every year [1]. Anti-disease immunity against malaria is not easily induced: in endemic areas this takes many years of repeated exposure to develop [2], and sterile protection against infection does not seem to be induced at all [3]. Also candidate vaccines have shown only limited protective efficacy so far [4], [5]. Novel vaccines and drugs can be tested for efficacy at an early stage of clinical development in Controlled Human Malaria Infection (CHMI) studies, exposing a small number of healthy volunteers to *Plasmodium falciparum* by bites from infected *Anopheles* mosquitoes. Immunization of healthy volunteers under chloroquine ChemoProphylaxis with Sporozoites (CPS-CQ immunization) efficiently, reproducibly and dose-dependently induces protection against homologous CHMI [6], [7], shown in a subset of volunteers to last for more than 2 years [8]. CPS-CQ immunization requires exposure to bites from only a total of 30–45 *P. falciparum* infected mosquitoes to induce 89–95% protection [6], [7], [9]. In contrast, protection by immunization with radiation-attenuated sporozoites (RAS) requires a minimum of 1000 infected mosquito bites [10], or intravenous injection of five times 135,000 cryopreserved sporozoites [11].

The unprecedented efficiency of the CPS immunization regime may relate to its design: in contrast to RAS, CPS immunization allows full liver stage development and exposure to early blood-stages. Moreover, chloroquine is known for its immunomodulatory capacities [12]–[14] that may play a role in induction of protection, which is mediated by pre-erythrocytic immunity [9] including antibodies directed against sporozoites [15]–[17], and likely T cells targeting liver-stages [7]. Next to chloroquine, mefloquine (MQ) is the only licensed drug for chemoprophylaxis that does not affect pre-erythrocytic stage development [18]. We therefore aimed to assess whether chloroquine could be replaced by mefloquine for CPS immunization. In a double blind randomized controlled clinical trial we assessed safety, immunogenicity and protection against challenge for CPS-MQ compared to CPS-CQ.

## Methods

### Study subjects

Healthy subjects between 18 and 35 years old with no history of malaria were screened for eligibility based on medical and family history, physical examination and standard hematological and biochemical measurements. Urine toxicology screening was negative in all included subjects; none of the subjects were pregnant or lactating. Serological analysis for HIV, hepatitis B, hepatitis C and *P. falciparum* asexual blood-stages was negative in all subjects. All subjects had an estimated 10-year risk smaller than 5% of developing a cardiac event as estimated by the Systematic Coronary Evaluation System adjusted for the Dutch population [19]. None of the subjects had travelled to a malaria-endemic area during or within 6 months prior to the start of the study. All subjects provided written informed consent before screening. The Central Committee for Research Involving Human Subjects of The Netherlands approved the study (NL 37563.058.11). Investigators complied with the Declaration of Helsinki and Good Clinical Practice including monitoring of data. This trial is registered at ClinicalTrials.gov, identifier NCT01422954. The protocol for this trial and supporting CONSORT checklist are available as supporting information (**Checklist S1** and **Protocol S1**).

### Study design and procedures

**Figure 1:**
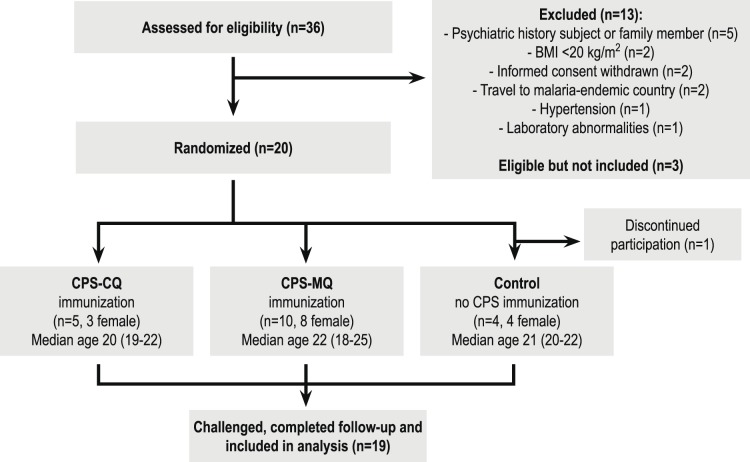
Study flow diagram. Thirty-six subjects were screened for eligibility, of whom twenty were included in the trial and randomized over three groups. One control subject was excluded after initiation of chemoprophylaxis but before the first immunization because of an unexpected visit to a malaria-endemic area during the study period. In a double-blind fashion, fifteen subjects received either CPS-CQ or CPS-MQ immunization and four control subjects received bites from uninfected mosquitoes and mefloquine prophylaxis. Subjects received a challenge infection by bites of five infected mosquitoes sixteen weeks after discontinuation of prophylaxis.

This single center, double blind randomized controlled trial was conducted at Leiden University Medical Center (Leiden, the Netherlands) from April 2012 until April 2013 (**Figure 1**). Twenty subjects were randomly divided into three groups by an independent investigator using a computer-generated random-number table. Subjects, investigators and primary outcome assessors were blinded to the allocation. Subjects in the CPS-CQ group (n = 5) received a standard prophylactic regimen of chloroquine consisting of a loading dose of 300 mg on the first and fourth day and subsequently 300 mg once a week for 12 weeks. Subjects in the CPS-MQ group (n = 10) and the control group (n = 5) received mefloquine prophylaxis starting with a loading split dose regimen to limit potential side-effects: 125 mg twice per week for a duration of 3 weeks and subsequently 250 mg once a week for 12 weeks. Chloroquine and mefloquine were administered as capsules, indistinguishable from each other. During this period all subjects were exposed to the bites of 8 *Anopheles* mosquitoes three times at monthly intervals, starting 22 days after start of mefloquine prophylaxis and 8 days after start of chloroquine prophylaxis. Volunteers in the CPS-CQ and CPS-MQ groups received bites from mosquitoes infected with the *P. falciparum* NF54 strain, control subjects received bites from uninfected mosquitoes. The immunization dose was based on our previous dose-de-escalation trial [7] and aimed to establish partial protection in the CPS-CQ group in order to enable detection of either improved or reduced protection in the CPS-MQ group. Sample sizes were calculated based on the expected difference of 4 days in prepatent period between the CPS-CQ and CPS-MQ groups, a standard deviation of 1.6 and 2.3 days respectively, an α of 5% and a power of 0.90. This calculation resulted in a CPS-CQ group of 4 and a CPS-MQ group of 8 subjects. To account for possible dropouts based on (perceived) side effects we included one and two extra volunteers in the CPS-CQ and CPS-MQ groups respectively. The control group was included as infectivity control for the challenge infection.

On days 6 to 10 after each immunization by mosquito exposure, all subjects were followed on an outpatient basis and peripheral blood was drawn for blood smears, standard hematological measurements, cardiovascular markers and retrospective qPCR.

Twenty weeks after the last immunization, sixteen weeks after discontinuation of prophylaxis, all subjects were challenged by the bites of five mosquitoes infected with the homologous NF54 *P. falciparum* strain, according to previous protocols [20]. After this challenge-infection, all subjects were checked twice daily on an outpatient basis from day 5 up until day 15 and once daily from day 16 up until day 21 for symptoms and signs of malaria. Thick blood smears for parasite detection were made during each of these visits after challenge, hematological and cardiovascular markers were assessed daily. As soon as parasites were detected by thick smear, subjects were treated with a standard curative regimen of 1000 mg atovaquone and 400 mg proguanil once daily for three days according to Dutch national malaria treatment guidelines. If subjects remained thick smear negative, they were presumptively treated with the same curative regimen on day 21 after challenge infection. All subjects were followed closely for 3 days after initiation of treatment and complete cure was confirmed by two negative blood smears after the last treatment dose. Chloroquine and mefloquine levels were measured retrospectively in citrate-plasma from the day before challenge by liquid chromatography (detection limit for both chloroquine and mefloquine: 5 µg/L) [21].

*Anopheles stephensi* mosquitoes for immunizations and challenge-infection were reared according to standard procedures at the insectary of the Radboud university medical center. Infected mosquitoes were obtained by feeding on NF54 gametocytes, a chloroquine- and mefloquine-sensitive *P. falciparum* strain, as described previously [22]. After exposure of volunteers, all blood-engorged mosquitoes were dissected to confirm the presence of sporozoites. If necessary, feeding sessions were repeated until the predefined number of infected or uninfected mosquitoes had fed.

### Endpoints

The primary endpoint was prepatent period, defined as the time between challenge and first positive thick blood smear. Secondary endpoints were parasitemia and kinetics of parasitemia as measured by qPCR, adverse events and immune responses.

### Detection of parasites by thick smear

Blood was sampled twice daily from day 5 until day 15 and once daily from day 16 up until day 21 after challenge and thick smears were prepared and read as described previously [9]. In short, approximately 0.5 µl of blood were assessed by microscopy and the smear was considered positive if two unambiguous parasites were seen.

### Quantification of parasitemia by qPCR

Retrospectively, parasitemia was quantified by real-time quantitative PCR (qPCR) on samples from day 6 until day 10 after each immunization and from day 5 until day 21 after challenge as described previously [23], with some modifications. Briefly, 5 µl Zap-Oglobin II Lytic Reagent (Beckman Coulter) was added to 0.5 ml of EDTA blood, after which the samples were mixed and stored at −80°C. After thawing, samples were spiked with the extraction control Phocine Herpes Virus (PhHV) and DNA was extracted with a MagnaPure LC isolation instrument. Isolated DNA was resuspended in 50 µl H_2_O, and 5 µl was used as template. For the detection of *P. falciparum*, the primers as described earlier [23] and the TaqMan MGB probe AAC AAT TGG AGG GCA AG-FAM were used. For quantification of PhHV the primers GGGCGAATCACAGATTGAATC, GCGGTTCCAAACGTACCAA and the probe Cy5-TTTTTATGTGTCCGCCACCATCTGGATC were used. The sensitivity of qPCR was 35 parasites/ml of whole blood.

### Adverse events and safety lab

Adverse events (AEs) were recorded as following: mild events (easily tolerated), moderate events (interfering with normal activity), or severe events (preventing normal activity). Fever was recorded as grade 1 (>37⋅5°C–38⋅0°C), grade 2 (>38⋅0°C–39⋅0°C) or grade 3 (>39⋅0°C). Platelet and lymphocyte counts were determined in EDTA-anti-coagulated blood with the Sysmex XE-2100 (Sysmex Europe GmbH, Norderstedt, Germany). D-dimer concentrations were assessed in citrate plasma by STA-R Evolution (Roche Diagnostics, Almere, The Netherlands).

### Immunological analyses

In order to assess cellular immune memory responses, peripheral blood mononuclear cell (PBMC) re-stimulation assays were performed as described previously [7]. PBMCs were collected, frozen in fetal calf serum containing 10% dimethylsulfoxide, and stored in vapor phase nitrogen before initiation of prophylaxis (baseline; B) and one day before the challenge infection (C-1).

After thawing, PBMCs were re-exposed *in vitro* to *P. falciparum*-infected red blood cells (*Pf*RBC) and incubated for 24 hours at 37°C in the presence of a fluorochrome-labeled antibody against CD107a. Uninfected red blood cells (uRBCs) were used as a negative control. During the last 4 hours of incubation, 10 µg/ml Brefeldin A and 2 µM Monensin were added, allowing cytokines to accumulate within the cells. As a positive control, 50 ng/ml PMA and 1 mg/ml ionomycin were added for the last four hours of incubation. After 24 h stimulation, cells were further stained with a viability marker and fluorochrome-labeled antibodies against CD3, CD4, CD8, CD56, γδ-T cell receptor, IFNγ and granzyme B (**Table S1** [7]). For each volunteer, cells from all time points were tested in a single experiment: thawed and stimulated on the same day and stained the following day. Samples were acquired on a 9-color Cyan ADP (Beckman Coulter) and data analysis was performed using FlowJo software (version 9.6.4; Tree Star). A representative example showing the full gating strategy is shown in **Figure S1.** Gating of cytokine-positive cells was performed in a standardized way by multiplying a fixed factor with the 75 percentile of the geometric Mean Fluorescent Intensity (MFI) of cytokine negative PBMCs for each volunteer, time point and stimulus. Responses to uRBC were subtracted from the response to *Pf*RBC for each volunteer on every time point.

Plasma for the assessment of malaria-specific antibodies was collected and stored at baseline (B), 27 days after the first immunization (I1; one day before the second immunization), 27 days after the second immunization (I2; one day before the third immunization), and one day before the challenge infection (C-1). Antibody titers were assessed as described previously [17]. In summary, serially diluted citrate plasma was used to perform standardized enzyme-linked immunosorbent assay (ELISA) in NUNC Maxisorp plates (Thermo Scientific) coated with 1 µg/ml circumsporozoite protein (CSP), liver-stage antigen-1 (LSA-1) or merozoite surface protein-1 (MSP-1) antigen, diluted in PBS. Bound IgG was detected using horseradish peroxidase (HRP) conjugated anti-human IgG) (Thermo Scientific, 1/60000) and Tetramethylbenzidine (all Mabtech). Spectrophotometrical absorbance was measured at 450 nm. OD values were converted into AUs by four-parameter logistic curve fit using Auditable Data Analysis and Management System for ELISA (ADAMSEL-v1.1, http://www.malariaresearch.eu/content/software; accessed 27 October 2014). Levels of antibodies were calculated in relation to a pool of 100 sera from adults living in a highly endemic area in Tanzania (HIT serum [24]), which was defined to contain 100 arbitrary units (AU) of IgG directed against each antigen.

### Statistical analyses

The proportion of protected subjects in the CPS-CQ versus CPS-MQ group was tested with the Fisher’s exact test using Graphpad Quickcalcs online and the 95% confidence interval (CI) of protection for each group was calculated by modified Wald Method [25]. Further statistical analyses were performed with GraphPad Prism 5. Differences in prepatent period and time from qPCR positivity until thick smear positivity were tested by Mann Whitney test. Antibody levels are shown as individual titers with medians and differences between time points were analyzed by Friedman test with Dunn’s multiple comparison post-hoc test. Induction of cellular immune responses was tested for CPS-CQ and CPS-MQ groups separately by Wilcoxon matched-pairs signed rank test (B versus C-1). A p-value of <0.05 was considered statistically significant. Analyses of parasitemia were performed on log transformed data, the geometric mean peak parasitemia after each immunization was calculated using the maximum parasitemia for each subject.

## Results

### Safety of CPS-CQ and CPS-MQ immunization

**Figure 2:**
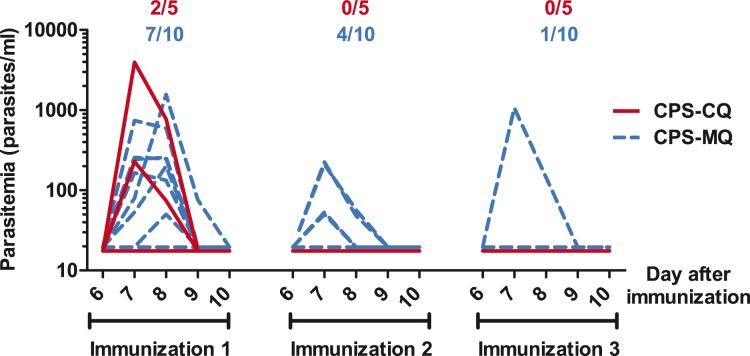
Parasitemia during CPS immunization. Parasitemia was determined retrospectively, once daily from day 6 until day 10 after each immunization, by real-time quantitative PCR (qPCR). Each line represents an individual subject from the CPS-MQ (dashed blue lines) or CPS-CQ group (red lines). The number of subjects with a positive qPCR/total number of volunteers in the CPS-MQ (blue) and CPS-CQ (red) groups after each immunization are shown above the graph. Values shown as 17.5 on the log-scale were negative (i.e. half the detection limit of the qPCR: 35 parasites/ml).

Twenty out of 36 screened subjects (median age 21 years; range 18–25) were included in the study (**Figure 1**). One control subject was excluded between start of prophylaxis and the first immunization because of an unexpected intermittent visit to a malaria-endemic area. Thick blood smears performed from day 6 up until day 10 after each immunization remained negative in all volunteers. As determined retrospectively by qPCR, 2/5 subjects in the CPS-CQ group and 7/10 subjects in the CPS-MQ group showed sub-microscopic parasitemia after the first immunization (geometric mean peak parasitemia for positive subjects: 948 parasites/ml [range 228–3938] and 256 parasites/ml [range 48–1559] respectively, **Figure 2**). After the second immunization, four CPS-MQ subjects showed sub-microscopic parasitemia (geometric mean peak parasitemia for positive subjects 104 parasites/ml [range 48–223]), while none of the CPS-CQ subjects showed parasitemia. After the third immunization, only one CPS-MQ subject showed parasitemia by qPCR (peak parasitemia 1059 Pf/ml).

**Figure 3:**
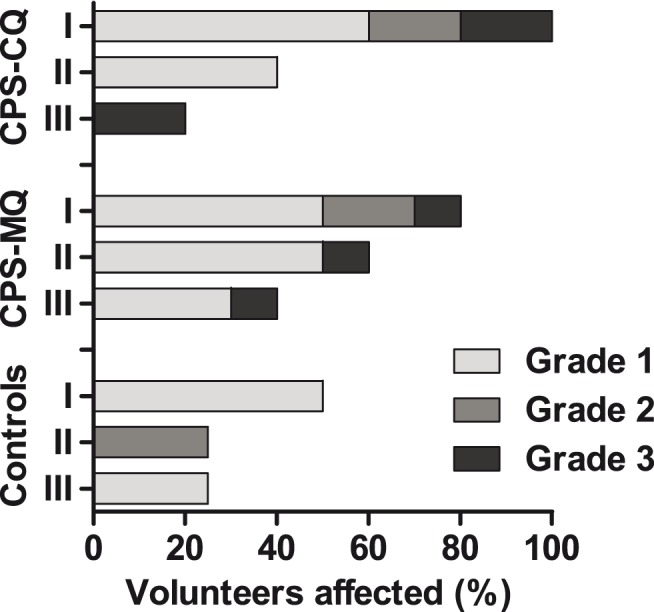
Adverse events during CPS immunization. Percentage of volunteers in each group experiencing possibly or probably related AE after the first (I), second (II) and third (III) immunization. AEs were evaluated at each visit and graded for severity as described in the methods paragraph: mild (light grey), moderate (dark grey) and severe (black). Only the highest intensity per subject is listed. No Serious Adverse Events occurred.

After the first immunization, all subjects (5/5) in the CPS-CQ group and almost all CPS-MQ subjects (8/9) experienced possibly or probably related AEs. One subject in each group had a grade 3 AE (headache and vomiting, respectively). Two control volunteers reported mild AEs (**Figure 3** and **Table S2**). After the second immunization, two CPS-CQ volunteers and six volunteers in the CPS-MQ group had mild AEs. Two control subjects experienced moderate and severe headache, respectively. After the third immunization, one volunteer in the CPS-CQ group and four CPS-MQ volunteers had AEs; one control subject experienced mild AEs (**Figure 3** and **Table S2**). One CPS-CQ subject reported moderate sleeping problems while taking chloroquine prophylaxis. One control subject had moderate problems with initiation of sleep and another control subject experienced vivid dreams under mefloquine prophylaxis. Other than mild to moderate dizziness and sleep related AEs, which all resolved after chemoprophylaxis was stopped, no neuropsychiatric AEs occurred. No serious adverse events occurred.

During immunization, one subject each in the CPS-CQ, CPS-MQ and control groups showed platelet counts below the lower limit of normal (150×10^9^/L); lowest values 105×10^9^/L, 116×10^9^/L and 131×10^9^/L, respectively. Three, five and two subjects from the CPS-CQ, CPS-MQ and control groups respectively, showed leukocyte counts below the lower limit of normal (4×10^9^/L); mean lowest value during immunization period: 3.8×10^9^/L [SD 1.2], 4.0×10^9^/L [SD 1.1] and 4.2×10^9^/L [SD 0.7] respectively. No subject developed leukocyte counts lower than 2.0×10^9^/L. One volunteer in each group showed leukocyte counts above the upper limit of normal (10×10^9^/L; highest values 10.8×10^9^/L, 13.8×10^9^/L and 10.1×10^9^/L respectively). After the first immunization, 3/5 CPS-CQ subjects, 7/10 in the CPS-MQ group and none in the control group developed elevated d-dimer levels (>500 ng/ml). After the second immunization, six CPS-MQ subjects but none in the CPS-CQ or control groups showed elevated d-dimer levels. After the third immunization, three CPS-MQ subjects showed elevated d-dimer levels, while none of the subjects in the other groups did.

### Protection against challenge infection

**Figure 4:**
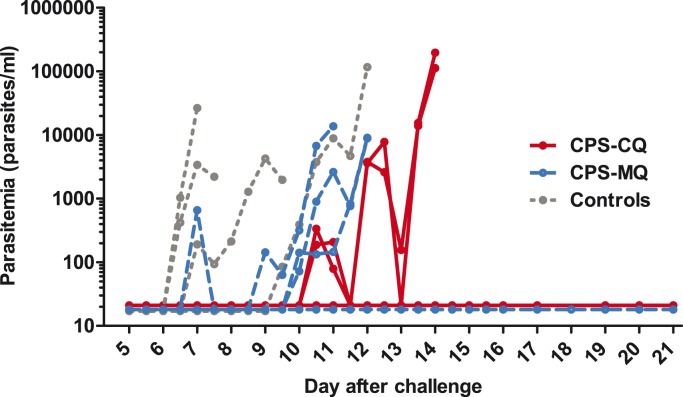
Parasitemia after challenge infection. Parasitemia was assessed retrospectively by real-time quantitative PCR (qPCR) twice daily from day 5 until day 15 and once daily up until day 21 after challenge. Each line represents an individual subject. Red lines represent CPS-CQ immunized volunteers (n = 5), dashed blue lines CPS-MQ immunized subjects (n = 10) and dotted grey lines malaria-naive control subjects (n = 4). Values shown as 17.5 on the log-scale were negative (i.e. half the detection limit of the qPCR: 35 parasites/ml).

In the CPS-CQ group 3/5 subjects and in the CPS-MQ group 7/10 volunteers were protected against challenge infection (Fisher’s exact test p = 1.0). All control subjects became thick smear positive (median day 8.5, range 7–12, p = 0.03 versus CPS-immunized subjects; **Table 1**). None of the protected subjects showed parasitemia by qPCR at any time point during follow-up (**Figure 4**). The median prepatent period was not significantly different between the CPS-CQ and CPS-MQ groups, neither when protected subjects were arbitrarily set at a prepatent period of 21 days (p = 1.00), nor when comparing unprotected subjects only (p = 0.1). The median chloroquine plasma concentration on the day before challenge infection was 9 µg/L (range 7–10) in the CPS-CQ group, and the median mefloquine concentration was 24 µg/L (range 5–116) in the mefloquine groups.

**Table 1:** Protection against challenge infection after CPS-CQ and CPS-MQ immunization.

Group	Protection			Unprotected volunteers					
				Day of positivity after challenge^c^					
	Number^a^	Percentage^b^	*p*	Thicksmear	*p*	qPCR	*p*	ΔTS+qPCR+^c^	*p*
CPS-CQ	3/5	60 (23–88)		14.0 (14.0–14.0)		11.3 (10.5–12.0)		2.8 (2.0–3.5)	
CPS-MQ	7/10	70 (39–90)	1.0^d^	12.0 (11.0–12.0)	0.10^f^	10.0 (9.0–10.0)	0.10^f^	2.0 (2.0–2.0)	0.40^f^
Control	0/4	0% (0–55)	0.03^e^	8.5 (7.0–12.0)	0.048 ^g^	6.3 (5.0–9.5)	0.056 ^g^	2.5 (1.5–2.5)	0.70 ^g^

a Presented as protected/total number of subjects.

b Presented as % protected (95% CI by modified Wald Method).

c Presented as median (range) days.

d,e p-value calculated by Fisher’s exact test comparing ^d^CPS-MQ versus CPS-CQ or ^e^control versus all CPS-immunized subjects.

f,g p-value calculated by Mann Whitney test comparing ^f^CPS-MQ versus CPS-CQ or ^g^control versus all CPS-immunized subjects (both excluding protected subjects).

### Immunogenicity of CPS-CQ and CPS-MQ

**Figure 5:**
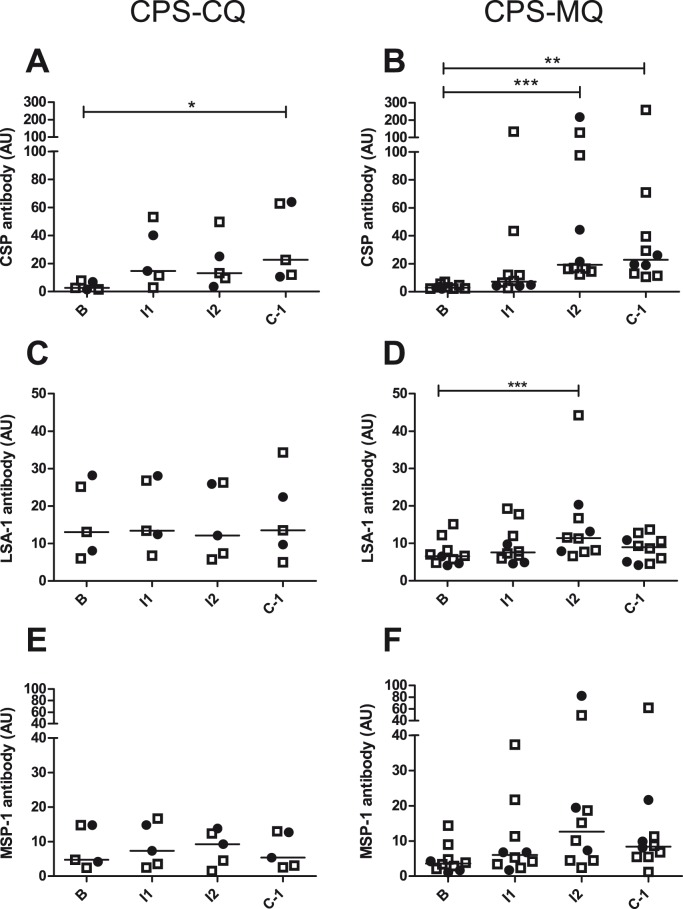
Antibody responses induced by CPS-CQ and CPS-MQ immunization. Antibodies against CSP (A and B; in AU), LSA-1 (C and D), and MSP-1 (E and F) were analyzed at baseline (B), 28 days after the first (I1) and second (I2) immunization and one day before challenge (C-1; 20 weeks after the last immunization) for all CPS-CQ (A, C and E, n = 5) and CPS-MQ (B, D and F, n = 10) immunized volunteers. Data are shown as individual titers with medians. Open squares indicate protected subjects, filled circles indicate unprotected subjects. Differences between the time points were analyzed by Friedman test with Dunn’s multiple comparison post-hoc test. Significant differences are indicated by asterices with * (p<0.05), ** (p<0.01), *** (p<0.001).

Antibodies against the pre-erythrocytic antigens CSP and LSA-1 and the cross-stage antigen MSP-1 were assessed by ELISA. Antibodies against CSP were induced in both CPS-CQ and CPS-MQ immunized volunteers (p<0.05 and p<0.01 respectively, on C-1; **Figure 5A and 5B**), but not significantly higher in protected compared to unprotected subjects (p = 0.88 and p = 0.48 respectively). Antibodies against LSA-1 were only significantly induced in CPS-MQ immunized volunteers on I2 (p<0.001; **Figure 5C and 5D**), although not higher in protected subjects (p = 0.39). Anti-MSP-1 antibodies by CPS immunization were not statistically significant increased in either group (**Figure 5E and 5F**).

**Figure 6:**
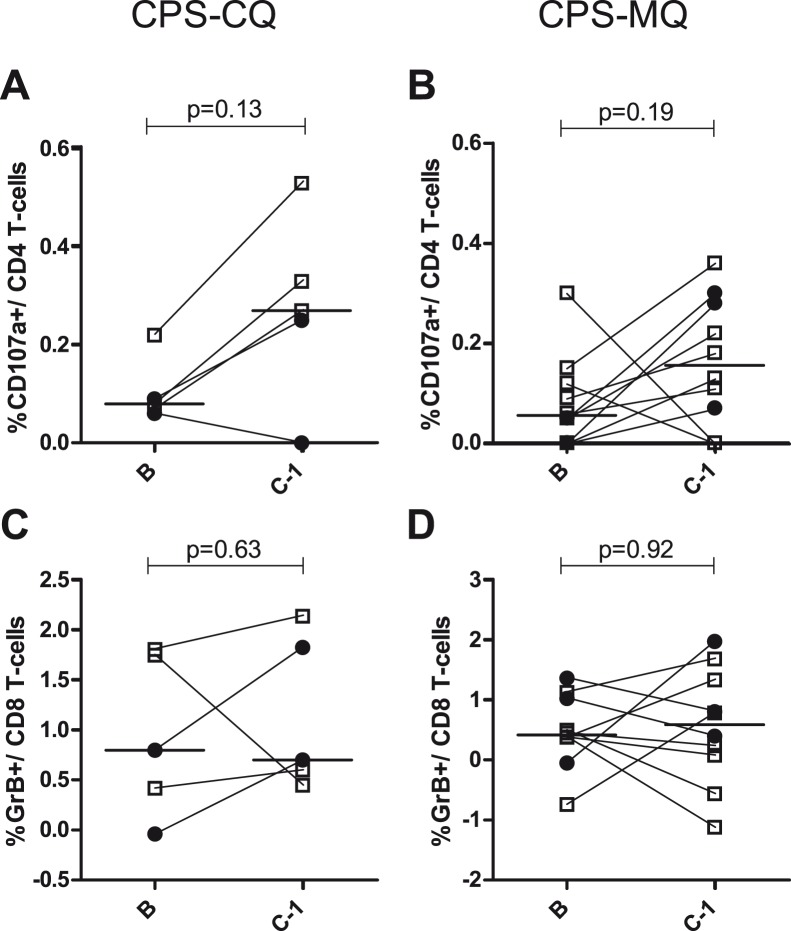
Cellular immune responses: CD107a expression by CD4 T cells and granzyme B production by CD8 T cells. CD107a expression by CD4 T cells after *Pf*RBC re-stimulation, corrected for uRBC background in CPS-CQ (A) and CPS-MQ (B) groups; granzyme B production by CD8 T cells after *Pf*RBC re-stimulation, corrected for uRBC background in CPS-CQ (C) and CPS-MQ (D) groups. Symbols and lines represent individual subjects before immunization (B) and one day before challenge (C-1). Open squares indicate protected subjects, filled circles indicate unprotected subjects. Differences between B and C-1 for all subjects were tested by Wilcoxon matched-pairs signed rank test.

IFNγ production by both adaptive and innate cell subsets in response to *in vitro P. falciparum* re-stimulation was induced by both CPS-CQ and CPS-MQ (**Figure S2**), without a clear quantitative or qualitative difference between the study groups. Next, CD107a expression by CD4 T cells and granzyme B production by CD8 T cells, both associated with protection in a previous CPS-CQ trial [7], were assessed by flow cytometry. Four out of 5 CPS-CQ and 8/10 CPS-MQ immunized subjects showed induction of CD107a expression by CD4 T cells upon *in vitro* re-stimulation after immunization (**Figure 6A and 6B**). Although volunteer numbers were too low to reach statistical significance, the magnitude of this response appeared to be associated with protection for CPS-CQ (**Figure 6A**), while for CPS-MQ it was not (**Figure 6B** **)**. Granzyme B production by CD8 T cells was not significantly induced in either CPS-CQ or CPS-MQ group, nor was it associated with protection (**Figure 6C and 6D**).

After challenge, MSP-1 specific antibodies were boosted in all unprotected volunteers (fold change median 20.4 (range 7.1–33.6), 76.0 (5.7–106.3) and 7.7 (2.9–15.3) for CPS-CQ, CPS-MQ and control groups respectively). None of the protected subjects showed an increase in MSP-1 antibody levels on C+35 compared to C-1 (median fold change 1.0 (range 1.0–1.3) and 1.0 (0.6–2.4) for CPS-CQ and CPS-MQ groups, respectively).

## Discussion

Immunization of healthy volunteers with *P. falciparum* sporozoites while taking mefloquine prophylaxis is safe, induces both humoral and cellular immune responses and protects against homologous malaria challenge.

Although most volunteers experienced AEs after the first immunization, their frequency declined after subsequent immunizations in line with a reducing number of volunteers developing parasitemia. The majority of AEs was mild, with only 10–20% of subjects experiencing a grade 3 AEs after each immunization. In general, the reported neurologic and psychiatric side effects of mefloquine are a major concern limiting its acceptability and clinical application. In this study, mild to moderate dizziness and sleep-related complaints occurred in a small number of subjects in both chloroquine and mefloquine groups. Although this study was not powered to detect differences in AEs, frequency of neuropsychiatric AEs did not appear to differ between both drugs. This is in line with most reports in literature comparing AEs of mefloquine or chloroquine (with or without proguanil) for chemo-prophylactic use [26]–[29] although one study found more neuropsychiatric AEs in subjects taking mefloquine by retrospective questionnaire [30]. Taking the small sample size into consideration, both CPS-CQ and CPS-MQ immunization regimens appear to be reasonably well tolerated and safe. In 2013, however, after completion of this study, the U.S. Food and Drug Administration (FDA) issued a boxed warning for mefloquine, stating that neurologic side effects might be permanent. This might lead to adjustment of prophylaxis guidelines and limitation of mefloquine use where alternatives are available, as for now it remains a recommended antimalarial prophylactic for several target groups [31].

In previous studies we showed that 19/20 subjects (95%) were protected after bites from 45 infected mosquitoes, 8/9 (89%) after bites from 30 and 5/10 (50%) after bites from 15 infected mosquitoes during chloroquine prophylaxis [6], [7], [9]. The 60–70% protection observed in the current CPS-CQ and CPQ-MQ groups, immunized with bites from 24 mosquitoes, demonstrates the reproducibility of CPS immunization and indicates a linear relationship between immunization dose and protection. This confirms the consistency of the CPS approach and is remarkable, given the assumed variation in the number of sporozoites injected by mosquitoes [32]. This study further establishes CPS immunization as a worthwhile immunization protocol to relatively easily induce protection and create differentially protected cohorts to study target antigens and correlates of protection, both of which would be highly valuable tools in the search for *P. falciparum* vaccines and biomarkers of protection [33].

Although the study was not powered to detect these differences, there are hints suggestive of more efficient induction of protection by CPS-CQ compared to CPS-MQ: i) the two unprotected CPS-CQ volunteers showed a longer prepatent period than the CPS-MQ subjects (14 versus 12 days, Mann-Whitney test p = 0.13); ii) induction of immunity required less immunizations in the CPS-CQ group i.e. none of these subjects showed blood-stage parasites after the second immunization while subjects in the CPS-MQ group still developed parasitemia after the second and third immunization. If there is a difference between CPS-CQ and CPS-MQ in protective efficacy, it is small, but possibly detectable in larger cohorts or when the immunization dose is further reduced.

Induction of anti-circumsporozoite antibodies by CPS-CQ is consistent with previous work, but neither anti-LSA-1, nor MSP-1 antibodies were induced by CPS-CQ in the current study [17]. Antibodies against the latter antigens are dose-dependently induced [17], and the current immunization regime using bites from 3×8 *P. falciparum*-infected mosquitoes might have been insufficient [7]. The induction of cellular *P. falciparum-*specific memory responses, as reflected by IFNγ production, is in line with previous CPS-CQ studies, even though limited sample size hampered statistical significance for some cell types. Interestingly, CD107a expression by CD4 T cells upon *in vitro* re-stimulation, associated with protection in a previous CPS-CQ study [7], appeared again to be associated with protection in the CPS-CQ group, but not the CPS-MQ group. Granzyme B production by CD8 T cells upon *in vitro* re-stimulation did not appear to be a reproducible marker of protection in this second CPS study [7]. Whether this might be related to immunization dose remains to be investigated in future CPS trials.

The striking efficiency of CPS immunization might at least be partly due to the established immune modulating properties of the 4-amino-quinoline chloroquine [12], possibly reflected by the more efficient induction of degranulating CD4 T cells. Chloroquine has been shown to increase cross-presentation in hepatitis B vaccination and influenza [13], [14], and thus may enhance cellular immune responses considered essential for protection against liver-stages [12]. For mefloquine, a 4-methanolquinoline, this immune-modulating property has, to our knowledge, not been reported. A possible strategy to assess whether chloroquine and/or mefloquine indeed have immune enhancing effects on whole sporozoite immunization would be to compare immunization with RAS in the presence or absence of these drugs.

Mefloquine or chloroquine plasma concentrations were still detectable in all volunteers one day before the challenge infection. Possible contributing effects of these remaining drug levels to the protective efficacy outcome were considered in several ways; i) The interval between first qPCR and thick smear positivity, as proxy for parasite multiplication, was 2.8 in the CPS-CQ group, 2.0 in the CPS-MQ group and 2.5 in the control group. This interval is similar to previous CHMI studies with the NF54 *P. falciparum* strain in the absence of prophylactic drug levels [7], [34]; ii) the two volunteers with the highest mefloquine levels (116 and 77 µg/L) were control subjects who became thick smear positive with only a minimal delay in patency within the time-frame of historical controls [35]; iii) plasma chloroquine and mefloquine levels at C-1 were in all volunteers well below the minimum therapeutic concentration (CQ: 30 µg/L [36]) or the concentration at which breakthrough infections are observed in non-immune people (MQ<406–603 µg/L [37]). iv) We cannot rule out that protected subjects experienced transient parasitemia after challenge, which was cleared in the first blood-stage cycle by remaining drug levels. But because parasitemia was not detected by qPCR in any of the protected subjects at any time point after challenge potential parasitemia must have been below the qPCR detection limit of 35 parasites/ml, indicating a reduction of at least 92% in liver load, given a geometric mean height of the first peak or parasitemia in non-immune historical controls of 456 parasites/ml [35]; v) None of the protected subjects showed a boost in anti-MSP-1 antibodies after challenge while all unprotected subjects did, suggesting that protected subjects did not experience blood-stage parasitemia after challenge. [9]. From these combined data we believe that remaining drug concentrations are unlikely to have contributed to the observed protection, although this cannot be formally excluded.

A review of rodent studies using different attenuation methods for whole sporozoite immunization shows that increased development of the parasite in the liver, but absence of blood-stage parasitemia during immunization is associated with the highest protective efficacy [38]. It would therefore be interesting to investigate CPS immunization with alternative antimalarials with varying targets in the parasite life cycle. CPS immunization with causal prophylactic drugs affecting liver-stages, e.g. primaquine, will likely results in a reduction of AEs because of reduced or absent blood-stage exposure. Whether antigen-exposure is sufficient to induce protection when the liver-stage is abrogated, remains to be answered.

In conclusion, we show that immunization of healthy volunteers under mefloquine prophylaxis with *P. falciparum* sporozoites is safe, immunogenic and protective. These findings could have important implications for malaria vaccine development and further development of CPS approaches.

## Supporting Information

Figure S1 **Gating strategy. **(**A**) Representative flow cytometry plots for a uRBC stimulated sample from one volunteer at baseline (before immunization). Singlet viable PBMCs were subdivided into (i) CD56hi NK cells, (ii) CD56dim NK cells, (iii) NKT cells, (iv) γδT cells, (v) CD8 T cells, (vi) CD4 T cells. (**B**) Gating of IFNγ, CD107a and granzyme B positive cells for uRBC, *Pf*RBC and PMA/ionomycin re-stimulated cells at baseline. For uRBC and *Pf*RBC stimulation CD4 T cells are shown, for PMA/ionomycin total viable PBMCs. Within each sample, gating of cytokine-positive cells was performed in a standardized way by multiplying a fixed factor with the 75 percentile of the geometric Mean Fluorescent Intensity (MFI) of cytokine negative PBMCs.
(TIF)

Figure S2 **Cellular immune responses: IFNγ production.** IFNγ production by different cell subtypes in response to *in vitro* re-stimulation with *Pf*RBC (corrected for uRBC background), before immunization (B) and one day before challenge (C-1). Differences between B and C-1 were tested by Wilcoxon matched-pairs signed rank test.
(EPS)

Table S1 **Antibodies used for flow cytometry.**
(DOC)

Table S2 **Possibly and probably related adverse events during CPS-CQ and CPS-MQ immunization.**
(DOC)

Protocol S1 **Trial protocol.**
(PDF)

Checklist S1 **CONSORT checklist.**
(PDF)
