# Supplementary figures and images for: Sporozoite Immunization of Human Volunteers under Mefloquine Prophylaxis Is Safe, Immunogenic and Protective: A Double-Blind Randomized Controlled Clinical Trial
Source: PLoS One. 2014 Nov 14;9(11):e112910. doi: 10.1371/journal.pone.0112910 (PMC4232459; doi:10.1371/journal.pone.0112910)

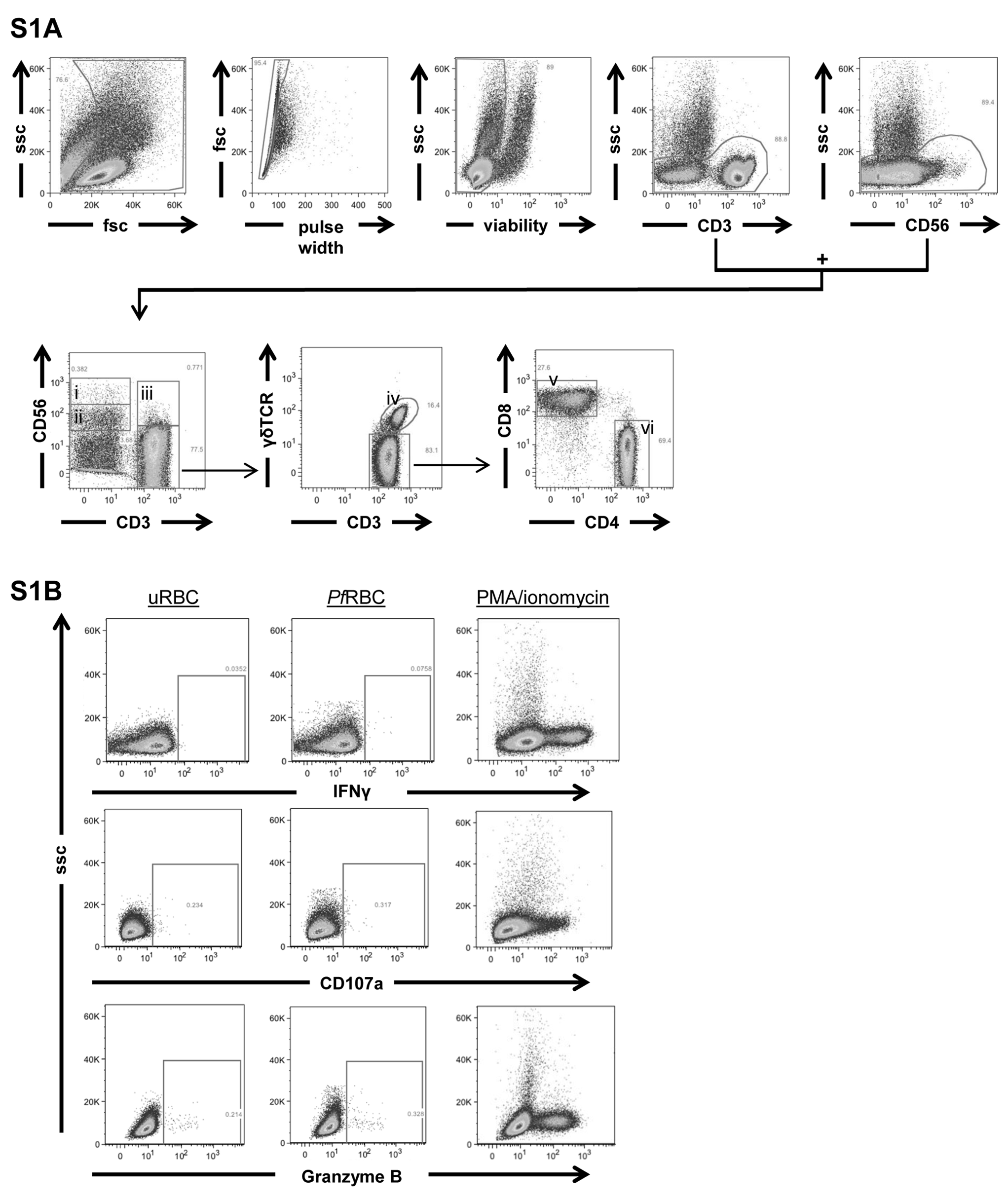

Supplement: Figure S1 — Gating strategy. (A) Representative flow cytometry plots for a uRBC stimulated sample from one volunteer at baseline (before immunization). Singlet viable PBMCs were subdivided into (i) CD56hi NK cells, (ii) CD56dim NK cells, (iii) NKT cells, (iv) γδT cells, (v) CD8 T cells, (vi) CD4 T cells. (B) Gating of IFNγ, CD107a and granzyme B positive cells for uRBC, PfRBC and PMA/ionomycin re-stimulated cells at baseline. For uRBC and PfRBC stimulation CD4 T cells are shown, for PMA/ionomycin total viable PBMCs. Within each sample, gating of cytokine-positive cells was performed in a standardized way by multiplying a fixed factor with the 75 percentile of the geometric Mean Fluorescent Intensity (MFI) of cytokine negative PBMCs. (TIF) [file pone.0112910.s001.tif]
