# Supplementary material for: Sporozoite Immunization of Human Volunteers under Mefloquine Prophylaxis Is Safe, Immunogenic and Protective: A Double-Blind Randomized Controlled Clinical Trial
Source: PLoS One. 2014 Nov 14;9(11):e112910. doi: 10.1371/journal.pone.0112910 (PMC4232459; doi:10.1371/journal.pone.0112910)
Supplement: Table S1 — Antibodies used for flow cytometry. (DOC) [file pone.0112910.s003.doc]

| **Target** | **Fluorochrome** | **Clone** | **Supplier** |
| --- | --- | --- | --- |
| Fixable viability | Aqua | N/A | eBioscience |
| CD3 | PerCP | UCHT-1 | Biolegend |
| CD4 | ECD | SFCI12T4D11 | BeckmanCoulter |
| CD8 | APC-H7 | SK1 | BD Biosciences |
| γδTcell receptor | PE | IMMU510 | BeckmanCoulter |
| CD56 | Biotin | HCD56 | Biolegend |
| Streptavidin | eF660 | N/A | eBioscience |
| IFNγ | PeCy7 | 4S.B3 | Biolegend |
| CD107a | Pacific Blue | H4A3 | Biolegend |
| Granzyme B | FITC | GB11 | Biolegend |

**Table S1. Antibodies used for flow cytometry.**
