# Supplementary material for: Sporozoite Immunization of Human Volunteers under Mefloquine Prophylaxis Is Safe, Immunogenic and Protective: A Double-Blind Randomized Controlled Clinical Trial
Source: PLoS One. 2014 Nov 14;9(11):e112910. doi: 10.1371/journal.pone.0112910 (PMC4232459; doi:10.1371/journal.pone.0112910)
Supplement: Table S2 — Possibly and probably related adverse events during CPS-CQ and CPS-MQ immunization. (DOC) [file pone.0112910.s004.doc]

|  |  | **CPS-CQ** | | | | **CPS-MQ** | | | | **Control** | | | |
| --- | --- | --- | --- | --- | --- | --- | --- | --- | --- | --- | --- | --- | --- |
|  |  | **CQ** | **I** | **II** | **III** | **MQ** | **I** | **II** | **III** | **MQ** | **I** | **II** | **III** |
| **Abdominal pain** | *None* | 5 (100) | 4 (80) | 5 (100) | 5 (100) | 10 (100) | 9 (90) | 10 (100) | 10 (100) | 2 (50) | 3 (75) | 4 (100) | 4 (100) |
|  | *Mild* | 0 (0) | 1 (20) | 0 (0) | 0 (0) | 0 (0) | 1 (10) | 0 (0) | 0 (0) | 1 (25) | 1 (25) | 0 (0) | 0 (0) |
|  | *Moderate* | 0 (0) | 0 (0) | 0 (0) | 0 (0) | 0 (0) | 0 (0) | 0 (0) | 0 (0) | 1 (25) | 0 (0) | 0 (0) | 0 (0) |
|  | *Severe* | 0 (0) | 0 (0) | 0 (0) | 0 (0) | 0 (0) | 0 (0) | 0 (0) | 0 (0) | 0 (0) | 0 (0) | 0 (0) | 0 (0) |
| **Arthralgia** | *None* | 5 (100) | 5 (100) | 5 (100) | 5 (100) | 10 (100) | 10 (100) | 10 (100) | 10 (100) | 4 (100) | 4 (100) | 4 (100) | 4 (100) |
|  | *Mild* | 0 (0) | 0 (0) | 0 (0) | 0 (0) | 0 (0) | 0 (0) | 0 (0) | 0 (0) | 0 (0) | 0 (0) | 0 (0) | 0 (0) |
|  | *Moderate* | 0 (0) | 0 (0) | 0 (0) | 0 (0) | 0 (0) | 0 (0) | 0 (0) | 0 (0) | 0 (0) | 0 (0) | 0 (0) | 0 (0) |
|  | *Severe* | 0 (0) | 0 (0) | 0 (0) | 0 (0) | 0 (0) | 0 (0) | 0 (0) | 0 (0) | 0 (0) | 0 (0) | 0 (0) | 0 (0) |
| **Chest pain** | *None* | 5 (100) | 5 (100) | 5 (100) | 5 (100) | 10 (100) | 10 (100) | 10 (100) | 10 (100) | 4 (100) | 4 (100) | 4 (100) | 4 (100) |
|  | *Mild* | 0 (0) | 0 (0) | 0 (0) | 0 (0) | 0 (0) | 0 (0) | 0 (0) | 0 (0) | 0 (0) | 0 (0) | 0 (0) | 0 (0) |
|  | *Moderate* | 0 (0) | 0 (0) | 0 (0) | 0 (0) | 0 (0) | 0 (0) | 0 (0) | 0 (0) | 0 (0) | 0 (0) | 0 (0) | 0 (0) |
|  | *Severe* | 0 (0) | 0 (0) | 0 (0) | 0 (0) | 0 (0) | 0 (0) | 0 (0) | 0 (0) | 0 (0) | 0 (0) | 0 (0) | 0 (0) |
| **Chills** | *None* | 5 (100) | 5 (100) | 5 (100) | 5 (100) | 10 (100) | 10 (100) | 10 (100) | 10 (100) | 4 (100) | 4 (100) | 4 (100) | 4 (100) |
|  | *Mild* | 0 (0) | 0 (0) | 0 (0) | 0 (0) | 0 (0) | 0 (0) | 0 (0) | 0 (0) | 0 (0) | 0 (0) | 0 (0) | 0 (0) |
|  | *Moderate* | 0 (0) | 0 (0) | 0 (0) | 0 (0) | 0 (0) | 0 (0) | 0 (0) | 0 (0) | 0 (0) | 0 (0) | 0 (0) | 0 (0) |
|  | *Severe* | 0 (0) | 0 (0) | 0 (0) | 0 (0) | 0 (0) | 0 (0) | 0 (0) | 0 (0) | 0 (0) | 0 (0) | 0 (0) | 0 (0) |
| **Diarrhea** | *None* | 5 (100) | 5 (100) | 4 (80) | 5 (100) | 10 (100) | 9 (90) | 10 (100) | 10 (100) | 4 (100) | 4 (100) | 4 (100) | 4 (100) |
|  | *Mild* | 0 (0) | 0 (0) | 1 (20) | 0 (0) | 0 (0) | 1 (10) | 0 (0) | 0 (0) | 0 (0) | 0 (0) | 0 (0) | 0 (0) |
|  | *Moderate* | 0 (0) | 0 (0) | 0 (0) | 0 (0) | 0 (0) | 0 (0) | 0 (0) | 0 (0) | 0 (0) | 0 (0) | 0 (0) | 0 (0) |
|  | *Severe* | 0 (0) | 0 (0) | 0 (0) | 0 (0) | 0 (0) | 0 (0) | 0 (0) | 0 (0) | 0 (0) | 0 (0) | 0 (0) | 0 (0) |
| **Dizziness** | *None* | 4 (80) | 5 (100) | 5 (100) | 5 (100) | 10 (100) | 9 (90) | 9 (90) | 9 (90) | 3 (75) | 4 (100) | 4 (100) | 4 (100) |
|  | *Mild* | 1 (20) | 0 (0) | 0 (0) | 0 (0) | 0 (0) | 0 (0) | 1 (10) | 0 (0) | 0 (0) | 0 (0) | 0 (0) | 0 (0) |
|  | *Moderate* | 0 (0) | 0 (0) | 0 (0) | 0 (0) | 0 (0) | 1 (10) | 0 (0) | 0 (0) | 1 (25) | 0 (0) | 0 (0) | 0 (0) |
|  | *Severe* | 0 (0) | 0 (0) | 0 (0) | 0 (0) | 0 (0) | 0 (0) | 0 (0) | 1 (10) | 0 (0) | 0 (0) | 0 (0) | 0 (0) |
| **Fatigue** | *None* | 5 (100) | 5 (100) | 5 (100) | 5 (100) | 10 (100) | 9 (90) | 10 (100) | 10 (100) | 4 (100) | 4 (100) | 4 (100) | 4 (100) |
|  | *Mild* | 0 (0) | 0 (0) | 0 (0) | 0 (0) | 0 (0) | 1 (10) | 0 (0) | 0 (0) | 0 (0) | 0 (0) | 0 (0) | 0 (0) |
|  | *Moderate* | 0 (0) | 0 (0) | 0 (0) | 0 (0) | 0 (0) | 0 (0) | 0 (0) | 0 (0) | 0 (0) | 0 (0) | 0 (0) | 0 (0) |
|  | *Severe* | 0 (0) | 0 (0) | 0 (0) | 0 (0) | 0 (0) | 0 (0) | 0 (0) | 0 (0) | 0 (0) | 0 (0) | 0 (0) | 0 (0) |
| **Fever** | *None* | 5 (100) | 4 (80) | 5 (100) | 5 (100) | 10 (100) | 7 (70) | 10 (100) | 8 (80) | 4 (100) | 4 (100) | 4 (100) | 4 (100) |
|  | *Mild* | 0 (0) | 0 (0) | 0 (0) | 0 (0) | 0 (0) | 3 (30) | 0 (0) | 1 (10) | 0 (0) | 0 (0) | 0 (0) | 0 (0) |
|  | *Moderate* | 0 (0) | 1 (20) | 0 (0) | 0 (0) | 0 (0) | 0 (0) | 0 (0) | 1 (10) | 0 (0) | 0 (0) | 0 (0) | 0 (0) |
|  | *Severe* | 0 (0) | 0 (0) | 0 (0) | 0 (0) | 0 (0) | 0 (0) | 0 (0) | 0 (0) | 0 (0) | 0 (0) | 0 (0) | 0 (0) |
| **Headache** | *None* | 3 (60) | 0 (0) | 4 (80) | 4 (80) | 7 (70) | 4 (40) | 5 (50) | 7 (70) | 4 (100) | 4 (100) | 3 (75) | 3 (75) |
|  | *Mild* | 2 (40) | 3 (60) | 1 (20) | 0 (0) | 3 (30) | 4 (40) | 4 (40) | 3 (30) | 0 (0) | 0 (0) | 0 (0) | 1 (25) |
|  | *Moderate* | 0 (0) | 1 (20) | 0 (0) | 0 (0) | 0 (0) | 2 (20) | 0 (0) | 0 (0) | 0 (0) | 0 (0) | 1 (25) | 0 (0) |
|  | *Severe* | 0 (0) | 1 (20) | 0 (0) | 1 (20) | 0 (0) | 0 (0) | 1 (10) | 0 (0) | 0 (0) | 0 (0) | 0 (0) | 0 (0) |
| **Malaise** | *None* | 5 (100) | 4 (80) | 5 (100) | 5 (100) | 10 (100) | 9 (90) | 10 (100) | 9 (90) | 4 (100) | 4 (100) | 4 (100) | 4 (100) |
|  | *Mild* | 0 (0) | 0 (0) | 0 (0) | 0 (0) | 0 (0) | 0 (0) | 0 (0) | 0 (0) | 0 (0) | 0 (0) | 0 (0) | 0 (0) |
|  | *Moderate* | 0 (0) | 1 (20) | 0 (0) | 0 (0) | 0 (0) | 1 (10) | 0 (0) | 1 (10) | 0 (0) | 0 (0) | 0 (0) | 0 (0) |
|  | *Severe* | 0 (0) | 0 (0) | 0 (0) | 0 (0) | 0 (0) | 0 (0) | 0 (0) | 0 (0) | 0 (0) | 0 (0) | 0 (0) | 0 (0) |
| **Myalgia** | *None* | 5 (100) | 4 (80) | 5 (100) | 5 (100) | 10 (100) | 10 (100) | 9 (90) | 9 (90) | 4 (100) | 4 (100) | 4 (100) | 4 (100) |
|  | *Mild* | 0 (0) | 1 (20) | 0 (0) | 0 (0) | 0 (0) | 0 (0) | 1 (10) | 1 (10) | 0 (0) | 0 (0) | 0 (0) | 0 (0) |
|  | *Moderate* | 0 (0) | 0 (0) | 0 (0) | 0 (0) | 0 (0) | 0 (0) | 0 (0) | 0 (0) | 0 (0) | 0 (0) | 0 (0) | 0 (0) |
|  | *Severe* | 0 (0) | 0 (0) | 0 (0) | 0 (0) | 0 (0) | 0 (0) | 0 (0) | 0 (0) | 0 (0) | 0 (0) | 0 (0) | 0 (0) |
| **Nausea** | *None* | 4 (80) | 4 (80) | 5 (100) | 5 (100) | 10 (100) | 7 (70) | 10 (100) | 10 (100) | 2 (50) | 4 (100) | 4 (100) | 4 (100) |
|  | *Mild* | 1 (20) | 1 (20) | 0 (0) | 0 (0) | 0 (0) | 2 (20) | 0 (0) | 0 (0) | 1 (25) | 0 (0) | 0 (0) | 0 (0) |
|  | *Moderate* | 0 (0) | 0 (0) | 0 (0) | 0 (0) | 0 (0) | 1 (10) | 0 (0) | 0 (0) | 1 (25) | 0 (0) | 0 (0) | 0 (0) |
|  | *Severe* | 0 (0) | 0 (0) | 0 (0) | 0 (0) | 0 (0) | 0 (0) | 0 (0) | 0 (0) | 0 (0) | 0 (0) | 0 (0) | 0 (0) |
| **Vomiting** | *None* | 5 (100) | 4 (80) | 5 (100) | 5 (100) | 10 (100) | 9 (90) | 10 (100) | 10 (100) | 4 (100) | 4 (100) | 4 (100) | 4 (100) |
|  | *Mild* | 0 (0) | 0 (0) | 0 (0) | 0 (0) | 0 (0) | 0 (0) | 0 (0) | 0 (0) | 0 (0) | 0 (0) | 0 (0) | 0 (0) |
|  | *Moderate* | 0 (0) | 1 (20) | 0 (0) | 0 (0) | 0 (0) | 0 (0) | 0 (0) | 0 (0) | 0 (0) | 0 (0) | 0 (0) | 0 (0) |
|  | *Severe* | 0 (0) | 0 (0) | 0 (0) | 0 (0) | 0 (0) | 1 (10) | 0 (0) | 0 (0) | 0 (0) | 0 (0) | 0 (0) | 0 (0) |
| **Vivid dreams or other sleeping problems** | *None* | 4 (80) | 5 (100) | 5 (100) | 5 (100) | 10 (100) | 10 (100) | 10 (100) | 10 (100) | 2 (50) | 3 (75) | 4 (100) | 4 (100) |
|  | *Mild* | 0 (0) | 0 (0) | 0 (0) | 0 (0) | 0 (0) | 0 (0) | 0 (0) | 0 (0) | 1 (25) | 1 (25) | 0 (0) | 0 (0) |
|  | *Moderate* | 1 (20) | 0 (0) | 0 (0) | 0 (0) | 0 (0) | 0 (0) | 0 (0) | 0 (0) | 1 (25) | 0 (0) | 0 (0) | 0 (0) |
|  | *Severe* | 0 (0) | 0 (0) | 0 (0) | 0 (0) | 0 (0) | 0 (0) | 0 (0) | 0 (0) | 0 (0) | 0 (0) | 0 (0) | 0 (0) |
| **Other** | *None* | 4 (80) | 4 (80) | 5 (100) | 5 (100) | 10 (100) | 4 (40) | 8 (80) | 8 (80) | 3 (75) | 4 (100) | 4 (100) | 3 (75) |
|  | *Mild* | 1 (20) | 1 (20) | 0 (0) | 0 (0) | 0 (0) | 4 (40) | 2 (20) | 2 (20) | 0 (0) | 0 (0) | 0 (0) | 1 (25) |
|  | *Moderate* | 0 (0) | 0 (0) | 0 (0) | 0 (0) | 0 (0) | 2 (20) | 0 (0) | 0 (0) | 0 (0) | 0 (0) | 0 (0) | 0 (0) |
|  | *Severe* | 0 (0) | 0 (0) | 0 (0) | 0 (0) | 0 (0) | 0 (0) | 0 (0) | 0 (0) | 1 (25) | 0 (0) | 0 (0) | 0 (0) |

**Table S2. Possibly and probably related adverse events during CPS immunization*^#^.**

*Number of volunteers (*%)*; for each subject, only the highest intensity is listed.

^#^CQ, MQ: after start of respective chemoprophylaxis up until first immunization; I, II, III: after the first, second and third immunization respectively.
