# Supplementary material for: Sporozoite Immunization of Human Volunteers under Mefloquine Prophylaxis Is Safe, Immunogenic and Protective: A Double-Blind Randomized Controlled Clinical Trial
Source: PLoS One. 2014 Nov 14;9(11):e112910. doi: 10.1371/journal.pone.0112910 (PMC4232459; doi:10.1371/journal.pone.0112910)
Supplement: Protocol S1 — Trial protocol. (PDF) [file pone.0112910.s005.pdf]

CLINICAL TRIAL PROTOCOL

Immunization with *Plasmodium falciparum* sporozoites under chloroquine versus mefloquine prophylaxis

Version 2.3, 29 May 2012
ZonMw2

PROTOCOL TITLE: "Immunization with *Plasmodium falciparum* sporozoites under chloroquine versus mefloquine prophylaxis"

**Protocol ID**	**ZonMw2**
**Short title**	**Immunization with *Pf* sporozoites under chloroquine versus mefloquine**
**Version**	**2.3**
**Date**	**29 May 2012**
**Coordinating investigator**	**Else M. Bijker, MD**<br>Tel +31 24 3619515<br>Fax :+31 24 3614666<br>e-mail: e.bijker@ncmls.ru.nl
**Principal investigator**	**Leo G. Visser, MD PhD**<br>Tel: +31 71 526 2613<br>Fax: +31 71 526 6758<br>e-mail: l.g.visser@lumc.nl
**Clinical investigators**	**Remko Schats, MD**<br>Tel +31 71 526 2613<br>e-mail: r.schats@lumc.nl<br>**Guido Bastiaens, MD**<br>Tel +31 24 3619515<br>Fax :+31 24 3614666<br>e-mail: G.Bastiaens@ncmls.ru.nl
**Sponsor:**	*Radboud University Nijmegen Medical Center*<br>*Department of Medical Microbiology*<br>**Prof. Robert W. Sauerwein, MD PhD**<br>Tel : +31 24 3610577<br>Fax :+31 24 3614666<br>e-mail: R.Sauerwein@mmb.umcn.nl
**Biological Evaluator:**	**Rob Hermsen, PhD**<br>Tel : +31 24 3613663<br>Fax :+31 24 3614666<br>email : R.Hermsen@ncmls.ru.nl
**Grant**	**ZonMw**
**Independent physician(s)**	**Frank P. Kroon, MD PhD**<br>Tel: +31 71 526 2620<br>Fax: +31 71 526 6758<br>e-mail: F.P.Kroon@lumc.nl
**Safety monitor**	**Mark G.J. de Boer, PhD**<br>Tel: +31 71 526 5475<br>Fax: +31 71 526 6758<br>e-mail: M.G.J.de_Boer@lumc.nl
**Laboratory sites**	*Department of Parasitology*<br>*Department of Medical Microbiology*<br>**Lisette van Lieshout, PhD**

	Tel: +31 71 5265062<br>e-mail: E.A.van_Lieshout@lumc.nl<br>**Jaco J. Verweij, PhD**<br>Tel: +31 71 5265062<br>e-mail: J.J.Verweij@lumc.nl
**Pharmacy**	*RUNMC Pharmacy*<br>**Head pharmacist: Marieke Welzen**<br>Tel: +31243616405<br>Fax:+31243668755<br>e-mail: M.Welzen@akf.umcn.nl<br>**Trial assistent: Sylvie Kok**<br>Tel: +31243616305<br>Fax: +31243668755<br>e-mail: Trial-Assist@akf.umcn.nl

PROTOCOL SIGNATURE SHEET

		Signature	Date
**Principal Investigator**	Leo G. Visser, MD PhD		
**Coordinating Investigator**	Else M. Bijker, MD		
**Sponsor**	Prof. Robert Sauerwein, MD PhD		

TABLE OF CONTENTS

LIST OF ABBREVIATIONS AND RELEVANT DEFINITIONS 7
SUMMARY 8
1. INTRODUCTION AND RATIONALE 10
1.1 Introduction 10
1.2 Rationale 10
1.3 Controlled Human Malaria Infections 12
1.4 Clinical Experience 12
1.5 Safety 12
2. OBJECTIVES 14
3. STUDY DESIGN 15
4. STUDY POPULATION 16
4.1 Population 16
4.2 Inclusion criteria 16
4.3 Exclusion criteria 16
4.4 Sample size calculation 17
5. TREATMENT OF SUBJECTS 18
5.1 Investigational product/treatment 18
5.2 Use of co-intervention 18
5.3 Escape medication 18
6. METHODS 19
6.1 Study parameters/endpoints 19
6.1.1 Main study parameter/endpoint 19
6.1.2 Secondary study parameters/endpoints 19
6.2 Randomisation, blinding and treatment allocation 20
6.3 Study procedures 21
6.3.1 Chloroquine or mefloquine prophylaxis 21
6.3.2 Immunization 22
6.3.3 Mosquito challenge 22
6.3.4 Treatment with Malarone® 22
6.3.5 Mosquito preparation 23
6.3.6 Blood sampling 23
6.3.7 Case report forms and data collection 24
6.3.8 Flow chart trial procedures 25
6.4 Withdrawal of individual subjects 28
6.5 Follow-up of subjects withdrawn from treatment 28
6.6 Premature termination of the study 28
7. SAFETY REPORTING 28
7.1 Section 10 WMO event 28
7.2 Adverse and serious adverse events 28
7.2.1 Adverse events 29
7.2.2 Serious adverse events 29

7.2.3 Adverse Event Data Collection ... 29
7.2.4 Assessment of causality ... 30
7.3 Follow-up of adverse events ... 30
7.1 Local Safety Monitor and Data Safety Monitoring Board (DSMB) ... 31
7.1.1 Review of Safety Data by the Safety Monitor and DSMB ... 31
8. STATISTICAL ANALYSIS ... 32
9. ETHICAL CONSIDERATIONS ... 33
9.1 Regulation statement ... 33
9.2 Recruitment and consent ... 33
9.3 Benefits and risks assessment, group relatedness ... 33
9.3.1 Ethical aspects concerning the production of *P. falciparum* infected mosquitoes ... 33
9.3.2 Ethical aspects concerning the use of human volunteers ... 33
9.4 Compensation for injury ... 34
9.5 Incentives ... 34
10. ADMINISTRATIVE ASPECTS AND PUBLICATION ... 34
10.1 Handling and storage of data and documents ... 34
10.2 Amendments ... 34
10.3 Annual progress report ... 34
10.4 End of study report ... 35
10.5 Public disclosure and publication policy ... 35
10.6 List of Publication and Authorship ... 35
11. REFERENCES ... 35

LIST OF ABBREVIATIONS AND RELEVANT DEFINITIONS

ABR	ABR form, General Assessment and Registration form, is the application form that is required for submission to the accredited Ethics Committee (In Dutch, ABR = Algemene Beoordeling en Registratie)
AE	Adverse Event
AR	Adverse Reaction
CA	Competent Authority
CCMO	Central Committee on Research Involving Human Subjects; in Dutch: Centrale Commissie Mensgebonden Onderzoek
CHMI	Controlled Human Malaria Infection
CV	Curriculum Vitae
DSMB	Data Safety Monitoring Board
EU	European Union
EudraCT	European drug regulatory affairs Clinical Trials
GCP	Good Clinical Practice
IB	Investigator's Brochure
IC	Informed Consent
IMP	Investigational Medicinal Product
IMPD	Investigational Medicinal Product Dossier
METC	Medical research ethics committee (MREC); in Dutch: medisch ethische toetsing commissie (METC)
Pf	Plasmodium falcuparum
(S)AE	(Serious) Adverse Event
SPC	Summary of Product Characteristics (in Dutch: officiële productinfomatie IB1-tekst)
Sponsor	The sponsor is the party that commissions the organisation or performance of the research, for example a pharmaceutical<br>company, academic hospital, scientific organisation or investigator. A party that provides funding for a study but does not commission it is not regarded as the sponsor, but referred to as a subsidising party.
SUSAR	Suspected Unexpected Serious Adverse Reaction
Wbp	Personal Data Protection Act (in Dutch: Wet Bescherming Persoonsgevens)
WMO	Medical Research Involving Human Subjects Act (in Dutch: Wet Medisch-wetenschappelijk Onderzoek met Mensen

SUMMARY

Rationale:

Malaria is one of the major infectious diseases in the world with a tremendous impact on the quality of life, significantly contributing to the ongoing poverty in endemic countries. It causes 800.000 deaths per year, the majority of which are children under the age of five. The malaria parasite enters the human body through the skin, by the bite of an infected mosquito. Subsequently, it invades the liver and develops and multiplies inside the hepatocytes. After a week, the hepatocytes burst open and the parasites are released in the blood stream, causing the clinical phase of the disease.

As a unique opportunity to study malaria immunology and efficacy of immunisation strategies, a protocol has been developed in the past to conduct controlled human malaria infections (CHMIs). CHMIs generally involve small groups of malaria-naïve volunteers infected via the bites of *P. falciparum* infected laboratory-reared *Anopheline* mosquitoes. Although potentially serious or even lethal, *P. falciparum* malaria can be radically cured at the earliest stages of blood infection when risks of complications are virtually absent.

We have shown previously that healthy human volunteers can be protected from a malaria mosquito (sporozoite) challenge by immunization with sporozoites (by mosquito bites) under chloroquine prophylaxis (CPS immunization). Interestingly, sterile protection in 100% of the human CPS immunized volunteers was achieved by a relatively miniscule dose, i.e. a total of 45 infectious mosquito bites, strikingly 20-fold more potent than the 1000 bites needed in a model using irradiated mosquitoes. One possible explanation for this efficiency is a contribution of the known immune modulating effects of chloroquine to the induction of protective immunity. We aim to assess this possible immune modulating effect in CPS immunization by comparing immunization with *P. falciparum* sporozoites under chloroquine with immunization under mefloquine prophylaxis, which has the same antimalarial effect, but not the immune modulating effects known from chloroquine.

Objectives:

Primary Objective: To compare protection against controlled *Plasmodium falciparum* malaria infection after immunization with sporozoites under chloroquine with immunization under mefloquine prophylaxis.

Secondary Objectives:

- To study and compare the development of parasitemia after challenge between study groups
- To analyze and compare the immune responses between study groups

Study design:

Single centre, double-blind randomized controlled clinical trial.

Study population:

A maximum of twenty healthy volunteers, aged 18 to 35 years, male and female, will participate in the study.

Intervention:

The study population will be randomly divided in three groups. For a period of 16 weeks, Group 1 (n=5) will receive chloroquine prophylaxis, Group 2 (n=10) and 3 (n=5) will receive mefloquine prophylaxis. In this period, all volunteers will be exposed to eight mosquito-bites at days 22, 50 and 78. Group 1 and 2 will receive *Plasmodium falciparum* infected mosquito-bites, Group 3 will receive uninfected mosquito-bites. Sixteen weeks after discontinuation of prophylaxis, all volunteers will be challenged by the bites of five *P. falciparum* infected mosquitoes. After challenge, all volunteers will be treated with a curative regimen of Malarone® (each tablet containing 250 mg atovaquone and 100 mg proguanil).

Study parameters/endpoints:

Primary endpoint:

Duration of prepatent period after challenge infection as measured by microscopy

Secondary endpoints:

- Parasitemia and kinetics of parasitemia as measured by PCR
- Frequency of signs or symptoms in study groups
- Immune responses between in study groups

Nature and extent of the burden and risks associated with participation, benefit and group relatedness:
Benefits: No benefit can be claimed for any of the volunteers. Even though immunized volunteers might be protected to *P. falciparum* from the challenge in this study, these effects may not apply to field situations. Therefore, volunteers will be advised to take regular malaria prophylaxis when travelling to malaria endemic areas in the future.
Risks: Risks for volunteers are related to exposure to (early) *P. falciparum* malaria infection and side-effects of chloroquine or mefloquine prophylaxis and Malarone® treatment.
Burden: The study is associated with an immunization period of three months in which the volunteers receive immunization with mosquito bites in every first week of the month, and have to make a visit to the trial centre five times in the second week. During these three months, they have to take weekly prophylaxis, either chloroquine or mefloquine. After the challenge there will be a short period (35 days) of intense clinical monitoring with frequent site visits and blood examinations. As it is unpredictable if and/or when subjects will develop a positive thick blood smear after challenge infection, it is impossible to state the exact number of site visits and blood examinations. However, the maximum number (in case a subject does not develop a positive blood smear) of site visits and blood examinations will be 65 with a maximum amount of collected blood of 1000 mL. In addition periodical physical examinations will be performed and the subject is asked to complete a diary.

1. INTRODUCTION AND RATIONALE

1.1 Introduction

Malaria is a common and serious tropical disease. It is a protozoan infection transmitted to human beings by mosquitoes. Human malaria is caused by five species of *Plasmodium* protozoa: *Plasmodium falciparum*, *P. vivax*, *P. ovale*, *P. malariae*, and *P. knowlesi*. Malaria is a public health problem in over 90 countries worldwide, inhabited by some 40% of the world population, i.e. over 2 billion people. It has been estimated that the incidence of malaria in the world is around 225 million clinical cases each year. People in tropical African countries account for more than 90% of these cases. Malaria mortality is estimated at 781.000 deaths worldwide per year. Most malaria deaths occur among young children in Africa, especially in remote rural areas with poor access to health services. Other high-risk groups include women during pregnancy and non-immune travellers, refugees, displaced persons, or labour forces entering into endemic areas. The epidemiology of malaria has been changing over recent years due to a combination of factors including increasing resistance of malarial parasites to chemotherapy and increasing insecticide resistance of the *Anopheles* mosquito vectors, ecological and climate changes and increased international travel to malaria-endemic areas.

Parasites (sporozoite stage) are injected into the skin capillaries by a female *Anopheles sp.* mosquito. From there they travel via the bloodstream to the liver, where they develop and multiply in liver cells before entering the blood stream again (merozoite stage) and invading red cells for further reproduction. Clinical malaria is caused by the cyclical proliferation of asexual stages in red blood cells. Malaria mortality is primarily due to organ dysfunction, in particular of the brain, following sequestration of infected red cells in the micro-vasculature.

There are several reasons why malaria continues to be one of the greatest health problems. One of the main reasons is difficulty with achieving adequate coverage with, and reducing pricing of, existing tools such as drugs and insecticide-treated bed nets. The decreasing effectiveness of existing tools (e.g. emergence of anti-malarial resistance by the parasite, resistance to insecticides by the mosquito vector, including pyrethroids) is a major challenge. The availability of an efficacious malaria vaccine would certainly be a major achievement to overcome the shortcomings of current control strategies. However, our understanding of the mechanisms underlying protective immunity is incomplete and specific markers of protection still need to be defined.

1.2 Rationale

Both natural and experimental exposure to malaria parasites can lead to development of protective immunity, providing a foothold for the development of a vaccine (Doolan 2009; Beeson 2008; Pombo 2002; Hoffman 2002; Roestenberg 2009). The clinical development of a malaria vaccine has been a continuous effort over the past half century (Epstein 2007) following the traditional vaccine development approach. Different formulations of a number of antigens and/or adjuvants have been tested in Phase I trials but only about a dozen candidates have been evaluated in Phase II clinical field trials (http://www.who.int/vaccine_research/links/Rainbow/en/index.html). No vaccine has worked well and most candidates have failed completely. The best vaccine to date, RTS,S, delays patency and reduces clinical severity, but does not provide long term protection against infection (Abdulla 2008; Breman 2009). Although a milestone in itself and potentially an additional tool in the combat against malaria, it is clear that better vaccine efficacies are required (Targett 2008).

A more effective alternative is immunization with sporozoites, attenuated by irradiation, which has been shown to induce strong protective immunity in rodents and in humans (Collins 1972; Hoffman 2002). Irradiation disrupts gene expression of sporozoites, which remain capable of hepatocyte invasion but do not complete liver-stage maturation and do not progress to the pathogenic blood stage (Silvie 2002). Cell-mediated immune responses against the liver stages of Plasmodium are responsible for this pre-erythrocytic immunity. However, induction of protective immunity in humans with this model requires a minimum of 1000 bites by irradiated mosquitoes during five or more immunization sessions, making practical application impossible. A technology platform to administer radiation attenuated sporozoites by syringe rather than mosquito bites is in progress but not yet available (Hoffman 2010).

A strikingly efficient induction of sterile protection against malaria can be achieved in rodents and humans by inoculation of intact sporozoites while concomitantly treating with chloroquine, a drug that kills parasites in the asexual blood stage but not in the pre-erythrocytic liver stage (CPS immunization; Belnoue 2004, Roestenberg 2009: see section K4a). Interestingly sterile protection in 100% of the human CPS immunized volunteers in a proof of concept study is achieved by a relatively miniscule dose, i.e. a total of 45 infectious mosquito bites, strikingly 20-fold more potent than the 1000 bites needed in the model using irradiated mosquitoes. This dose is also much lower than in malaria endemic areas where many years of natural exposure to infected mosquitoes are needed to achieve protection against malaria. Furthermore, this protection has been shown to last for 28 months (Roestenberg 2011), which is much longer than generally recorded after natural exposure. However, the underlying mechanisms of protective immunity induced by CPS immunization remain unclear.

Potent cellular immunity seems to be crucial to obtain protective pre-erythrocytic immunity. This study aims to investigate the possible role of chloroquine in the efficient induction of protection by CPS immunization and study the cellular immune responses.

Chloroquine has several immune modulating properties, which might enhance the induction of protective immunity. The direction of the immune system towards a more anti-inflammatory response, improvement of cross-presentation and inhibition of toll-like receptor (TLR)-9 mediated signaling in plasmacytoid dendritic cells are likely mechanisms of action (Sauerwein 2010).

TLR-9 is located in the endosomal compartment. Chloroquine interferes with endosomal acidification, which might be a mechanism by which chloroquine blocks signalling by this TLR. TLR-9 signalling is very relevant in the context of malaria because hemozoin, a degradation product of parasite-infected red blood cells, stimulates dendritic cells in a TLR-9 dependent way (Coban 2005), consequently increasing the regulatory T cell population (Hisaeda 2008). These regulatory T cells are inversely correlated with memory T cell responses (Walther 2009). Therefore, chloroquine might suppress this immune evading mechanism by inhibiting TLR-9 signalling, and therefore contribute to effective induction of protective immunity.

Moreover, chloroquine may improve cross-presentation and therefore the induction of a cytotoxic T lymphocyte (CTL) response. This is of potential importance in malaria, as a CD8+ T cell response is thought essential for the development of protective immunity to the liver stage of Plasmodium. Short-course treatment of mice with chloroquine improves the priming of naïve CD8+ T cell responses against soluble antigens in vivo (Garulli 2008). Cross-presentation of soluble viral antigens to specific CD8+ T cell clones by dendritic cells is greatly improved when they are pulsed with the antigen in the presence of chloroquine, which prevents endosomal acidification, and seems to promote the transfer of endocytosed material into the cytosol. Moreover, chloroquine administration to humans receiving a vaccine based on the hepatitis B virus envelope protein substantially increased the specific CTL response (Accapezzato 2005). The net result of cross-presentation depends on the routing and processing conditions such as acidification of the endosomal compartment, modulated by chloroquine (Belizaire 2009). In conclusion, chloroquine is an established immune modulating drug, routinely used clinically in auto-immune diseases with documented immune modulating properties (Kyburz 2006).

To explore the immune modulating effects of chloroquine on induction of protection and immune responses, we will compare the outcome following immunization with three times eight live *P. falciparum* infected mosquito bites under a prophylactic regime of either chloroquine or mefloquine. Both these structurally related registered anti-malarial drugs act on blood stage but not on liver stage parasites.

In order to show a potential difference between immunization under chloroquine versus mefloquine prophylaxis, we need an immunization dose on the edge of the required dose to confer protection. This dose (number of infected mosquito-bites) is based on the results of the ZonMw1 trial (NL33904.091.10). In this trial, eight out of nine volunteers were protected from a challenge infection after immunization with three times ten infected mosquito bites under chloroquine prophylaxis. In the group that was immunized with three times fifteen mosquito-bites under chloroquine prophylaxis however, four out of five volunteers were protected. This might mean that the immunization was slightly less potent this time compared to a previous trial (Roestenberg 2009). Therefore, three

times ten mosquito-bites might induce 100% protection in a next study. We therefore chose to immunize with three times eight infected mosquito bites.
In vitro- studies have shown an inhibiting effect of mefloquine on neutrophil function and on IL-2 production of lymphocytes after stimulation with mitogens or malaria-specific antigens (Labro 1988, Bygbjerg 1987, Bygbjerg 1986). However, all these effects occur at concentrations much higher than therapeutic plasma levels. One in vitro study from 1986 shows an inhibiting effect on NK cell activity of both chloroquine and mefloquine (Pedersen 1986). In conclusion, the known immune modulating effects of mefloquine are minimal at best and never used for this clinical application, in contrast to chloroquine. Therefore we do not expect an influence by mefloquine in this study.

1.3 Controlled Human Malaria Infections

Controlled human malaria infections are well accepted as a powerful tool for the evaluation of parasite development in humans. Parasite multiplication is the key parameter in such trials, which implies that parasitemia should be followed over a sufficiently lengthy period. For determination of both erythrocytic and pre-erythrocytic parasite multiplication, accurate measurement of parasitemia after exposure to infectious bites is absolutely essential. We have the experience and infrastructure to conduct controlled human malaria infections. We have also developed a very sensitive method of parasite detection by Real-time Quantitative PCR (RTQ-PCR) that will allow us to detect small differences in parasite density. Using these sensitive measurements of parasitemia, we have developed a statistical model of parasitemia after mosquito bite (Hermsen 2004). We will use our model to calculate numbers of infected liver cells and parasite multiplication factors in human volunteers over time.

1.4 Clinical Experience

There is a large clinical experience with infecting humans by the bite of *P. falciparum* sporozoite-infected mosquitoes. Since 1986 more than 1300 volunteers are challenged by the bites of mosquitoes fed on cultures of *P. falciparum* gametocytes to produce sporozoites (Chulay 1986). This has proved to be a reproducible, predictable and safe method of inducing *P. falciparum* malaria. The results of such studies were summarized in 1997 (Church 1997), in 2007 (Epstein 2007) and in 2011 (Roestenberg 2011, in press).
Controlled human malaria infections have been conducted in the Center for Clinical Malaria Research (CCMS) Nijmegen in over 150 volunteers since its origin in 2001. Controlled human malaria infections have also been conducted in 45 volunteers in Leiden in collaboration with the Center for Clinical Malaria Research (CCMS) Nijmegen since 2009. Standard operating procedures according to international standards are in place for both clinical and laboratory activities at both sites.

1.5 Safety

In February 2008 a cardiac SAE (CCMO08.1096/MA/14715) in a 20 year old female participating in a LSA3/Alhydrogel (LSA-3 CMO-07/37; NL14715.000.06) malaria vaccine trial was reported to the CCMO. The findings have been published as a case report entitled “Cardiac complication after controlled human malaria infection: case report”, A.E. Nieman et al, Malar. J. 2009 Dec 3;8(1):277 (See section K4b). The true nature and patho-physiological explanation of the event remain unclear. Following this event, recommendations of the European Malaria Vaccine Development Association and the CCMO to ensure maximal safety of participating human volunteers have been integrated in the malaria challenge protocol:

1. Riamet® is no longer used as treatment for experimentally induced malaria. It is replaced by Malarone®.
2. Volunteers with 1st or 2nd degree relatives with cardiac events under the age of 50 will be excluded from participation.
3. The occurrence of the cardiac SAE is included as part of the volunteer information sheet.
4. Volunteers will be required to stay at very close distance to the LUMC to ensure maximal safety from day 5 after challenge, until treatment has been finished (maximum 20 days).

5. Negative urine toxicology screening test is added as inclusion criterion.
6. Monitoring of highly sensitive troponin T (hsTropT), D-dimer, lactate dehydrogenase (LDH), thrombocytes, ADAMTS13 and fragmentocytes.

Since above mentioned adaptations, three more experimental malaria studies have been concluded in a total of 44 volunteers (NL30350.058.09, NL24193.091.09 and NL31858.091.10).

No cardiovascular adverse events occurred in any of the volunteers. Monitoring of cardio-vascular markers showed the following results:

Twenty-six out of thirty-one volunteers with a positive thick smear developed elevated d-dimer levels. Eight volunteers had elevated d-dimer levels without a positive thick smear. Median d-dimer peak concentration was 2410 ng/ml (n=30), range 540-14600 ng/ml. In all cases, d-dimer values normalized during follow-up.
ADAMTS13 and fragmentocytes were investigated in fifteen cases. The number of fragmentocytes was always within the normal range. ADAMTS13 approached abnormal values (38%) in one volunteer, but normalized activity was noticed in the follow-up period.

One serious adverse event occurred in a female volunteer in the TIP2 trial (NL31858.091.10). She reported chest pain the day after start of atovaquone/proguanil treatment for *Pf* parasitemia. Based on medical history, the chest pain when first seen was considered possibly consistent with angina pectoris. It resolved within one hour without treatment. She was admitted to the cardiac care unit for monitoring for 6.5 hours. The EKG showed a negative T-wave in V2, which had been absent at screening, but could have been caused by positioning of the electrodes. Subsequent EKG's were comparable to baseline, with a negative T in V1 only. Highly sensitive troponin T (detection limit 0.003 µg per liter) did not show any changes at the time of chest pain, six hours later, 17 hours later, daily for three days and at trial day 28 and 35. The trial was put on hold pending discussion with the Sponsor, the Safety Monitoring Committee (SMC), and the U.S. F.D.A. Since there was no more chest pain or changes in the EKG during the subsequent three days, the SMC considered the event not to be cardiac, and recommended the trial be resumed according to protocol within three days of the event.

Currently, two trials are being conducted in which the same safety parameters are being measured (NL33904.091.10 en NL34273.091.10). In one of these trials (ZonMw1, NL33904.091.10), HS-Troponin T values were slightly elevated (0.047 µg/L) in one asymptomatic volunteer just after extremely intensive sports activity. The DSMB of this trial concluded that this elevation in HS Troponin did not indicate cardiac damage, but was due to the sports activity and therefore this should not have consequences for the volunteer or the trial.
Otherwise, no abnormal HS-Troponin T values occurred in any of the studies.

2. OBJECTIVES

Primary Objective: To compare protection against controlled *Plasmodium falciparum* malaria infection after immunization with sporozoites under chloroquine with immunization under mefloquine prophylaxis.

Secondary Objectives:

- To study and compare the development of parasitemia after challenge between study groups
- To analyze and compare the immune responses between study groups

3. STUDY DESIGN

The study is a single centre, double-blind randomized controlled clinical trial. Volunteers, the investigators and the laboratory personnel will be blinded.
A maximum of 20 volunteers will be divided into three groups as shown in Table 1. All volunteers will receive weekly chloroquine or mefloquine prophylaxis for a period of 16 weeks (105 days). During these 16 weeks, on days 22, 50 and 78 groups 1 and 2 will be exposed to the bites of eight infected mosquitoes. Group 3 will receive an equal number of uninfected mosquito-bites.
If one of the volunteers is not fit to participate in the study on day -1, he or she will be replaced by an alternate volunteer who passed screening. For this purpose 3 additional volunteers will be screened for possible back-up. Fifteen weeks after discontinuation of prophylaxis, all 20 volunteers will be challenged (day 218) by the bites of 5 infectious mosquitoes (according to previous protocols).

**Group number**	**Prophylaxis**	**Immunization with infected mosquito-bites**
1 (n=5)	Chloroquine	Yes
2 (n=10)	Mefloquine	Yes
3 (n=5)	Mefloquine	No

Table 1, study groups.

A summary of the study schedule is shown in figure 1.

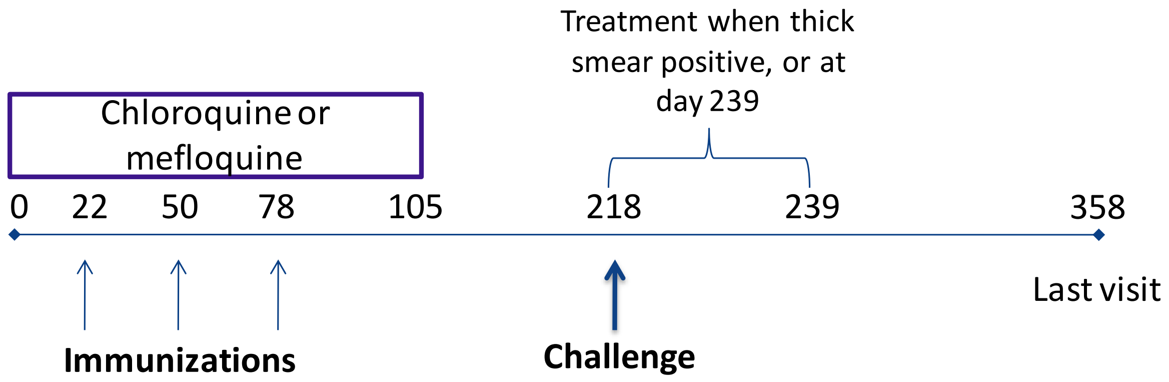

Figure 1: study schedule

4. STUDY POPULATION

4.1 Population

Twenty healthy, malaria naïve volunteers will be recruited to participate in the study. Advertisements will be placed in prominent places on different University campuses and other public places as well as on the intranet of the institutions. Furthermore, a facebook-page showing the advertisement-text will be designed to inform people about the trial. This brief advertisement (section E3) will indicate a telephone number to call and an email address for contact to request further information. It will furthermore indicate a website which contains a form. A general, short questionnaire will be completed over the phone, over email or by the form on the website. When seemingly suitable, volunteers will be invited to an information meeting, in which the study is explained to them by the study investigator, and given the information sheet (section E1), the application form (section F1a) and the insurance text (section E4) to study them at home. During and after the meeting there will be room for questions. Volunteers willing to apply fill in the application form and are invited to come to the screening visit. At this screening visit, the informed consent form (section E2) will be signed, the questionnaire answers will be discussed and inclusion and exclusion criteria will be checked. Also, a letter for the general practitioner will be signed and sent after screening (section K6a). Again, there will be room for questions. The possibility of withdrawal from the study, at any time and without any declaration of the reason will be pointed out to the volunteers. If volunteers prove to be eligible, they will be invited to the next visit.

4.2 Inclusion criteria

1. Age ≥ 18 and ≤ 35 years healthy volunteers (males or females)
2. Good health based on history and clinical examination
3. Negative pregnancy test
4. Use of adequate contraception for females
5. Signing of the informed consent form, thereby demonstrating understanding of the meaning and procedures of the study
6. Agreement to inform the general practitioner and to sign a request to release medical information concerning contra-indications for participation in the study
7. Willingness to undergo a *Pf* controlled infection through mosquito bites
8. Agreement to stay in a hotel room close to the trial center during a part of the study (Day 5 after challenge till treatment is finished)
9. Reachable (24/7) by mobile phone during the whole study period
10. Available to attend all study visits
11. Agreement to refrain from blood donation to Sanquin or for other purposes, during the whole study period
12. Willingness to undergo HIV, hepatitis B and hepatitis C tests
13. Negative urine toxicology screening test at screening visit and the day before challenge
14. Willingness to take a prophylactic regime of chloroquine or mefloquine and curative regimen of Malarone®

4.3 Exclusion criteria

1. History of malaria
2. Plans to travel to malaria endemic areas during the study period
3. Plans to travel outside of the Netherlands during the challenge period
4. Previous participation in any malaria vaccine study and/or positive serology for *Pf*
5. Symptoms, physical signs and laboratory values suggestive of systemic disorders including renal, hepatic, cardiovascular, pulmonary, skin, immunodeficiency, psychiatric, and other conditions which could interfere with the interpretation of the study results or compromise the health of the volunteers

6. History of diabetes mellitus or cancer (except basal cell carcinoma of the skin)
7. History of arrhythmias or prolonged QT-interval
8. Positive family history in 1st and 2nd degree relatives for cardiac events < 50 years old
9. An estimated, ten year risk of fatal cardiovascular disease of ≥5%, as estimated by the Systematic Coronary Risk Evaluation (SCORE) system
10. Clinically significant abnormalities in electrocardiogram (ECG) at screening
11. Body Mass Index (BMI) below 20 or above 30 kg/m2
12. Any clinically significant deviation from the normal range in biochemistry or hematology blood tests or in urine analysis
13. Positive HIV, HBV or HCV tests
14. Participation in any other clinical study within 30 days prior to the onset of the study
15. Enrollment in any other clinical study during the study period
16. For women: pregnancy or lactation
17. Volunteers unable to give written informed consent
18. Volunteers unable to be closely followed for social, geographic or psychological reasons
19. History of drug or alcohol abuse interfering with normal social function
20. A history of treatment for psychiatric disease or moderate or severe psychological episode in volunteer
21. A history of convulsions in volunteer
22. Severe depression, anxiety disorder of psychosis in first or second degree family
23. Contra-indications to Malarone®, chloroquine or mefloquine including hypersensitivity or treatment taken by the volunteer that interferes with Malarone®, chloroquine or mefloquine
24. The use of chronic immunosuppressive drugs, antibiotics, or other immune modifying drugs within three months of study onset (inhaled and topical corticosteroids and oral anti-histaminic are allowed) and during the study period
25. Any confirmed or suspected immunosuppressive or immunodeficient condition, including asplenia
26. Co-workers or trainees of the departments of Medical Microbiology, Parasitology, or Internal Medicine of the Leiden University medical Centre
27. A history of sickle cell anemia, sickle cell trait, thalassemia, thalassemia trait or G6PD deficiency

4.4 Sample size calculation

Our primary objective is to determine whether immunization with sporozoites under mefloquine confers equal protection against a controlled malaria infection as immunization with sporozoites under chloroquine prophylaxis. We expect a difference in prepatent period of 4 days between the positive control group (group 1, chloroquine immunization) and the experimental group (group 2, mefloquine immunization). In the experimental group we expect a standard deviation of 2.3 days, in the control groups we expect a standard deviation of 1.6 days, based on previous studies. Therefore, to show a difference in prepatent period of 4 days, we need an experimental group size of 8 volunteers and control groups of 4 volunteers (α = 5%, power 0.90).
As a result of side-effects from mefloquine and possibly perceived side-effects in the chloroquine group, we expect a drop-out of maximum 20% in all groups (Overbosch 2001). Therefore, we need an experimental group of 10 and control groups of 5 volunteers.

5. TREATMENT OF SUBJECTS

5.1 Investigational product/treatment

There is no investigational product in this study.

5.2 Use of co-intervention

All volunteers will receive weekly chloroquine or mefloquine prophylaxis according to a standard prophylactic regime as described in 6.3.1. All volunteers who undergo a challenge will be treated with Malarone® (tablets containing 250 mg atovaquon and 100 mg of proguanil) as described in section 6.3.4.

5.3 Escape medication

Volunteers are advised to take tripelennamine crème for the local treatment of mosquito bites. Volunteers are advised to take paracetamol for complaints secondary to the mosquito challenge (fever, muscle aches, headache, etc.). Tripelennamine crème, paracetamol or any other symptomatic treatment will be supplied to the volunteers. The maximum dose of paracetamol is 4 grams a day.

6. METHODS

6.1 Study parameters/endpoints

6.1.1 Main study parameter/endpoint

- Duration of prepatent period after challenge infection as measured by microscopy

Thick smear samples will be taken from a 3 ml EDTA vacutainer tube. Thick smears will be performed on all visits following immunizations and challenge infection until treatment is finished.
Thick smears will be performed according to a standard operating procedure which is based on a internationally harmonized protocol for thick smears in CHMIs (Moorthy et al., WHO). In short, 15µl of whole blood will be distributed on standardized 3-well slides, providing an equal slide thickness for all smears. Slides are dried and coloured with Giemsa staining. Per slide, 200 fields will be read. Slides are considered positive if they contain 2 or more parasites per 200 fields. Thick smear evaluation will take place at the LUMC.

6.1.2 Secondary study parameters/endpoints

- Development of parasitemia as measured by PCR
- Frequency of signs or symptoms in study groups
- Immune responses between study groups

Samples for RTQ-PCR will be collected from the same 3 ml EDTA vacutainer tubes as the thick smear sample. RTQ-PCR will be performed according to standard procedure described in Hermsen et al. Mol, Biochem. Parasitol. 2001; 118: 247-251. In short, RTQ-PCR will be performed on the multicopy 18S ribosomal RNA gene. All samples are spiked with murine white blood cells and a murine albumin gene PCR is used to determine efficacy of DNA isolation.
Samples for quantitative measurement of parasitemia will be prepared and stored at LUMC. Measurement by RTQ-PCR will be performed retrospectively at the department of Parasitology of the LUMC in collaboration with the Radboud University Nijmegen Medical Center, department of Medical Microbiology.

Signs and symptoms will be recorded at all visits, and whenever a trial volunteer reports signs or symptoms to the trial physician between visits. The following signs and symptoms will be solicited: Fever, Headache, Malaise, Fatigue, Myalgia, Arthralgia, Nausea, Vomiting, Chills, Diarrhoea, Abdominal pain (Verhage 2005), Chest pain, Palpitations and Shortness of breath. For more information on recording of adverse events please refer to section 7.2.

We aim to answer the following immunological questions:
a) Is there a difference between the acquisition of adaptive immune responses under chloroquine versus mefloquine prophylaxis?
b) How does immunization with sporozoites under chloroquine versus mefloquine prophylaxis alter innate immune cell activation (dendritic cells, natural killer (T)-cells, γδT cells)?
c) Is there a set of biomarkers that predicts effective induction of protective immunity by immunization with sporozoites under chloroquine or mefloquine prophylaxis?
d) How do immune transcriptome profiles differ during immunization under CQ versus MQ prophylaxis and is there a RNA response signature during immunization that correlates with protection from a challenge infection?

To answer these questions, several immunological assays will be performed. The choice for specific assays will

depend on results which are currently being obtained on samples from previous studies, but will most likely consist of the following:

- Phenotyping of peripheral blood mononuclear cells (T cells, B cells, regulatory T cells and innate cells) by flow cytometry
- Functional assays (antigen specificity):
    - T-cell stimulation assay (flow cytometry and/or ELISpot)
    - B-cell functional assay (ELISpot and ELISA)
- mRNA expression analysis by dual-color Reverse-Transcriptase Multiplex Ligation-dependent Probe Amplification (dcRT-MLPA)
- RNA transcriptome profiling by whole mRNA sequencing (collaboration with P. Crompton at NIAID, NIH, E. Kirkness at the J. Craig Venter Institute, LUMC and RUNMC)

Immunological endpoints will be measured at only a restricted number of time points during the study. In order to answer questions a) and b), PBMC's will be collected on day -1 (baseline), day 21 (day before first immunization), day 29 (day 7 after first immunization), day 32 (day 10 after first immunization), day 49 (before second immunization), day 57 (day 7 after second immunization), day 60 (day 10 after second immunization), day 77 (before third immunization), day 217 (pre-challenge), day 253 (post-treatment) and day 358 (follow-up) (see flow chart, section 6.3.8).
In order to answer questions c) and d), RNA samples will be collected on day -1, day 21, day 27, day 28, day 31, day 49, day 56, day 59, day 77, day 84, day 87, day 217, day 223, day 224, day 227, day of treatment, day 253 and day 358.

6.1.3 Exploratory study parameter/endpoint

- Metabolic responses between study groups

We want to investigate metabolic responses of volunteers during CPS immunization and after challenge infection. By using novel analyzing methods (time resolved analysis) of individual metabolic phenotypes (Suhre 2011 Nature) we want compare the metabolome of protected and unprotected individuals after immunization with *Plasmodium falciparum* sporozoites.
Serum left over from blood samples taken for study safety assessments will be collected at predefined time points once daily at regular visits. No additional blood is collected. Leftover serum will be collected from laboratory (CKCL LUMC) and stored at -80°C in two aliquots containing each maximum 1 ml of serum.
Urine samples will be collected in order to answer the question whether this less invasive strategy allows monitoring of metabolic responses. Two ml of urine will be stored at -80°C in two aliquots of 1 ml on the following time points: day -1, day 21, day 28, day 217, day of treatment and day 253.

6.2 Randomisation, blinding and treatment allocation

The 20 volunteers will be allocated to one of the three groups at random according to a randomization list. No stratification will be performed. The randomisation list will be prepared by an independent investigator at the RUNMC. The code can be broken by one of the investigators, always after discussion with the safety monitor, or by the safety monitor himself in case of an adverse event needing urgent treatment depending on the group the volunteer has been allocated to. The safety monitor may break the code if it is absolutely necessary for the monitoring of the safety of the volunteers. If the code is broken it will be documented and reported to the IRB.
On the day before the infection the required number of infected and uninfected mosquitoes will be placed in separate small cages with red or green labels respectively. These coloured labels will be covered with a neutral label with the volunteer code, in order to blind the volunteers and the attending clinician.

6.3 Study procedures

All volunteers will be closely followed as outpatients. For the duration of intensive monitoring (day 5 post challenge until day 3 after treatment) they will be required to stay in the study hotel, close to the Leiden University Medical Center. For the hotel period, volunteers are allowed to leave the hotel for daily activities, but will be required to stay at the hotel overnight for every night. They will receive breakfast at the hotel and one warm meal a day at the hospital canteen. Hotel and meal costs will be paid by the investigators. From day 5 till day 15 after challenge, volunteers will visit the trial centre two times a day. Since the likelihood of thick smear positivity drops significantly after the 15th day, de visit frequency is reduced to once daily from day 16 till day 21 after challenge. Volunteers are instructed to call the trial physicians at any time if they experience symptoms or complaints. The trial physician can decide to initiate additional diagnostics or treatment at all times. Table 6.3.8 shows the study procedures per visit.

Before the screening visit, volunteers will be asked to complete an application form which includes a questionnaire regarding their health (section F1b). All volunteers must consent with an HIV, hepatitis B, hepatitis C and toxicology test at screening. Female volunteers will be subjected to a urine pregnancy test at screening and the day before the challenge. All volunteers will repeat the urine toxicology test the day before challenge. At almost all visits, volunteers are subjected to blood withdrawal by venapuncture. All volunteers are asked to complete a diary (section F2).

The immunizations with mosquito bites and the mosquito challenge will be performed at the Central Animal Laboratory Facility, of the RUNMC, Nijmegen, Netherlands. The biological safety parameters will be measured on serum samples at the central laboratory of the Leiden University Medical Center. Assessment of blood slides will be performed by trained technicians of the department of Medical Microbiology of the Leiden University Medical Center. The immunological assessment will be performed both at the department of Medical Microbiology at the Nijmegen Center of Molecular Life Sciences of the RUNMC in Nijmegen and at the department of Infectious Diseases and Parasitology of the Leiden University Medical Center.

6.3.1 Chloroquine or mefloquine prophylaxis

The RUNMC pharmacy will prepare capsules containing chloroquine, mefloquine or placebo. The capsules will be indistinguishable from each other, and will be labelled with the volunteer number.
Group 1 will receive weekly chloroquine prophylaxis according to the standard prophylactic regime: a loading dose of 300 mg on day 14 and day 17 and then 300 mg once a week, starting on day 21, for a total duration of 13 weeks. On day 0, day 3, day 7 and day 10, group 1 will receive a placebo.
Group 2 and 4 will receive mefloquine prophylaxis, starting with a loading regime of split doses during the first three weeks: 125 mg on day 0, day 3, day 7, day 10, day 14 and day 17 and 250 mg once a week from day 21 onwards for a total duration of 13 weeks. The first three doses of mefloquine are split in order to reduce side-effects. This is a standard prophylactic regime as recommended by the Dutch LCR (Landelijk coördinatiecentrum reizigersadvisering) guidelines (LCR Malariabulletin 2011).

**Group**	**Day 0**	**Day 3**	**Day 7**	**Day 10**	**Day 14**	**Day 17**	**Day 21 – day 105 weekly**
**1. Chloroquine innmunization**	placebo	placebo	placebo	placebo	300 mg	300 mg	300 mg
**2. Mefloquine immunization**	125 mg	125 mg	125 mg	125 mg	125 mg	125 mg	250 mg

**3. Mefloquine control**	125 mg	125 mg	125 mg	125 mg	125 mg	125 mg	250 mg

Table 2. Prophylaxis regimes

Mefloquine and chloroquine are both registered for malaria prophylaxis. In this study, we use these drugs for this registered purpose. Despite a negative public perception on mefloquine, large pharmacoepidemiological studies have shown that serious adverse events are rare, and occur mostly at therapeutic concentration, not at prophylactic doses. A US evaluation of serious events (hospitalization data) found no association between mefloquine prescriptions and serious adverse events across a wide range of outcomes including mental disorders (Schlagenhauf 2010). The incidence of neuropsychological adverse events such as depression, strange dreams and insomnia is equal in mefloquine compared to chloroquine (Steffen 1993, Lobel 1993). Because a low BMI is an independent risk factor for neuropsychiatric events, we increased the minimum BMI from 18 to 20 kg/m^2 as inclusion criterion. Furthermore, we apply stringent inclusion criteria concerning psychiatric history and family history for psychiatric disease and split the doses in the first three weeks.

6.3.2 Immunization

On the day of immunization, the volunteers will be exposed to the feeds of 8 *Anopheles stephensi* mosquitoes by interrupted feeding. The first immunization will be performed on day 22.

6.3.3 Mosquito challenge

On the challenge day, all volunteers will be exposed to 5 infectious mosquitoes. The infection will be performed by placing a box containing mosquitoes on the forearm of the volunteer. (For more information concerning the production of mosquitoes, please refer to section 'Mosquito preparation' under 6.3.5). Mosquito feeding will be allowed for 10 minutes. Volunteers will receive a local treatment (tripelennamine crème) for mosquito bites and will be observed for 15 minutes after the feed. Directly after the feed, the mosquitoes will be dissected by a technician of the mosquito unit. This will be done to assure the presence of sporozoites in the salivary glands of the mosquitoes. Exposure will be repeated until the exact number of infected mosquito bites has been reached.
As long as there are volunteers present in the mosquito unit, there will be supervision of one of the clinical investigators. Another clinical investigator will then be on call, in case of emergency. Emergency aid kits will be present and readily available at any location, whenever there are volunteers present.

6.3.4 Treatment with Malarone®

All volunteers will be treated with Malarone® based on the following criteria:
1. Positive thick blood smear during regular check-up
2. Complaints of malarial infection and thick blood smear positive
3. By decision of study doctor or the safety monitor
4. On request of the volunteer
5. On day 21 post challenge, if the volunteer has remained thick blood smear negative
6. When hs Troponine T (Roche) > 0.1 µg/ml or on recommendation of the cardiologist
7. When thrombocytes < 75 x 10^9/l
8. Dependent on values for LDH, D-dimer, ADAMTS13 and fragmentocytes according to the following scheme:

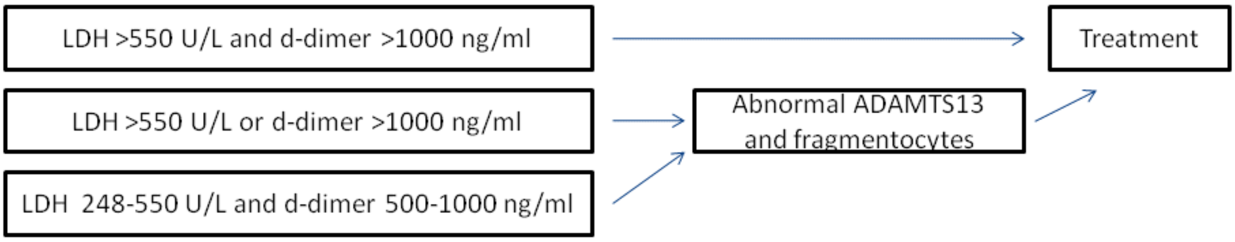

The treatment will consist of the drug Malarone® (atovaquon/proguanil). Dosing will be as follows: once daily 4 tablets of 250/100mg, during three days according to Dutch SWAB guidelines. This drug has been chosen because of its fast clinical response and the few side-effects. Furthermore, it has not been reported to have any cardiac side-effect. During treatment, complaints of malaria infection will be treated symptomatically. In addition to specific treatment with Malarone®, symptomatic treatment will be administered at the discretion of the attending physician.

Volunteers will not be admitted to the hospital during this study, unless the study doctors or the safety monitor deem it necessary, or on request of the volunteer.

D-dimer serum concentrations will be assessed by STA-R (Roche, Almere, The Netherlands). STA-R is a fully automated open random access coagulation analyser for clotting, chromogenic and immunological latex based coagulation tests. D-dimer concentrations are determined according to the manufacturer's instructions using a immunoturbidimetrical method, and are expressed in ng/mL (lower detection level 220 ng/mL upper detection level 5000 ng/mL).

6.3.5 Mosquito preparation

The culture of parasites and infection of mosquitoes has been a routine procedure for over 10 years now in the Malaria Unit of the Central Animal Facility of the RUNMC, Nijmegen. The isolate used in this study will be the NF54 isolate. The NF54 isolate originates from the Schiphol area and is sensitive to all antimalarial drugs (chloroquine, mefloquine, atovaquone/proguanil and arthemeter/lumefantrine).
NF54 is originally derived from patient material and cultured in vitro in RPMI-1640 medium with 10% serum and 5% haematocrit red blood cells. Both the serum and the red blood cells are obtained from the Nijmegen department of the Sanquin Bloedbank region Zuid-Oost, which is negative for malaria and seronegative for HIV, HBsAg, HCV, HTLV I+II and Lues. The cultures are checked for bacterial contamination, for mycoplasma and for blood transmitted diseases (HIV, HBV, HCV, HTLV 1+2).
To produce infectious gametocytes, the asexual parasites will be cultured in vitro. After 14 days of culture, the sexual stage parasites are obtained and prepared for feeding to 3-5 days old, and laboratory cultured *Anopheles stephensi* mosquitoes via a "membrane feeder". The percentage infected mosquitoes will be assessed after 7-10 days and after 14-17 days (see Product Information, D2a).

6.3.6 Blood sampling

During the study, blood samples will be drawn, for screening, safety and research purposes. The blood sampling schedule in the flow-chart section 6.3.8 shows the maximum amounts of blood that will be drawn. The following safety and screening tests will be performed:

- Haematology: Full Blood Count and differentiation of white cells, at V1 (screening), V2 (inclusion), V7-12, V15-19, V22-26 (control visits after immunizations), V5, V13, V20 and V27 (days before immunizations and challenge), daily during challenge period (day 223-239) until 3 days after treatment and at day 246 and 253.
- Biochemistry: Sodium, Potassium, Creatinin, ureum, ASAT, ALAT, AF, γGT, total bilirubin, at V1 (screening), V2 (inclusion), V27 (day before challenge), day T+2 and day 253. Glucose and total cholesterol only for screening purposes.
- Highly sensitive Troponin T, d-dimer and LDH at V1 (screening), V2 (inclusion), V7-12, V15-19 and V22-26 (control visits after immunizations), V27 (day before challenge), daily during challenge period (day 223-239) until 3 days after treatment and at day 246.

- Fragmentocytes and ADAMTS13 if there are abnormal results for d-dimer and LDH according to the scheme in section 6.3.4.

6.3.7 Case report forms and data collection

All data collected by the investigator is reported in case report forms. These forms, together with the investigator's notes are considered source data. Since all subjects will be healthy, there is no medical file for the study subjects, with exception of the medical file in case of adverse events/reactions resulting in a medical consultation or hospitalization. In this case the medical file will be considered as the source data. The diaries, produced by the study volunteers are also considered source data. They will be kept as source document in the investigator clinical file.

6.3.8 Flow chart trial procedures

	Screening visit	Inclusion	Start prophylaxis			First Immunization			Second Immunization			Third immunization	
	Number of visits fixed												
**Visit Number**	V1	V2	V3	V4	V5	V6	V7-12	V13	V14	V15-19	V20	V21	V22-26
**Trial timeline (D=day)**	± D-14	D-1	D0	D7	D21	D22	D27-32	D49	D50	D56-60	D77	D78	D84-88
**Frequency of visits**	1x	1x	1x	1x	1x	1x	1x	1x	1x	1x	1x	1x	1x
**Eligibility criteria + informed consent**	X												
**Demographic data, Medical history**	X												
**Physical examination**	X	X			X[1]		X[1]	X[1]		X[1]	X[1]		X[1]
**ECG**	X												
**Vital signs**	X	X	X	X	X		X	X		X	X		X
**Immunization with mosquito bites**						X			X			X	
**Challenge with 5 infected mosquitoes**													
**Collecting (serious) adverse events**			X	X	X	X	X	X	X	X	X	X	X
**Prophylaxis**			X	X	X	X	X	X	X	X	X	X	X
**Malarone® treatment**													
**Haematology tests[2] (3 ml)**	X	X			X		X	X		X	X		X
**Biochemistry tests[3] (3 ml)**	X	X											
**Glucose (2 ml)**	X												
**Serology[4] (6 ml)**	X												
**Parasitology[5] (3 ml)**		X				X	X	X		X	X		X
**Cellular/humoral immune response[6]**		X			X		X[7]	X		X[7]	X		
**Pregnancy and toxicology urine test**	X	X											
**Urine sample metabolomics**		X			X		X[11]						
**hs Trop T, LDH, d-dimer**	X	X					X			X			X
**Reviewing diaries**		X	X	X	X		X	X		X	X		X
**Safety report**							X			X			X

	Day before challenge	Challenge							End of study
	Number of visits fixed		Number of Visits depending on day of thick smear positivity		Number of visits fixed				
**Visit Number**	V27	V28	V29-50	V51-55	V56	V57-62	V63	V64	V65
**Trial timeline (D=day)**	D217	D218	D223-233	D234-238	TD[8] or D239	TD+1-3	D246	D253	D358
**Frequency of visits**	1x	1x	2x	1x	1x	2x	1x	1x	1x
**Eligibility criteria + informed consent**									
**Demographic data**, **Medical history**									
**Physical examination**	X		X[1]	X[1]	X[1]	X[1]	X	X[1]	X[1]
**ECG**	X								
**Vital signs**	X		X	X	X	X	X	X	X
**Immunization with mosquito bites**									
**Challenge with 5 infected mosquitoes**		X							
**Collecting (serious) adverse events**	X	X	X	X	X	X	X	X	X
**Prophylaxis**									
**Malarone® treatment**					X	X			
**Haematology tests[2] (3 ml)**	X		X*	X	X	X	X	X	
**Biochemistry tests[3] (3 ml)**	X					X (TD+2)		X	
**Glucose (2 ml)**									
**Serology[4] (6 ml)**									
**Parasitology[5] (3 ml)**	X		X**	X	X	X (DT+3)[9]	X	X	X
**Cellular/humoral immune response[6]**	X							X	X
**Pregnancy and toxicology urine test**	X								
**Urine sample metabolomics**	X				X			X	
**hs Trop T, LDH, d-dimer**	X		X	X	X	X	X		
**Reviewing diaries**	X		X	X	X	X	X	X	
**Safety report**			X[10]		X			X	

D = day, V = visit, TD = treatment day
*: once daily; **: twice daily

1: On indication
2: Hb, haematocrit, MCV, MCH, MCHC, platelets, WBC + differentiation

3: Creatinin, urea, sodium, potassium, bilirubin, AF, γGT, ASAT, ALAT, additional at screening: total cholesterol + glucose
4: HIV, HBV, HCV, Pf
5: Thick smear, PCR
6: Plasma collected from tubes for cellular assays will be stored for humoral assays
7: Immunological evaluation on day 29, 32, 57 and 60
8: TD: day of thick smear positivity and/or start treatment Malarone®
9: Thick smear at TD+3, if not negative treatment until twice negative
10: Safety evaluation on day 228
11: Urine sample for metabolomics on day 28

6.4 Withdrawal of individual subjects

Subjects can leave the study at any time for any reason if they wish to do so without any consequences. The investigator can decide to withdraw a subject from the study for urgent medical reasons.
Volunteers can be withdrawn from the study procedures for the following reasons:

- Any serious adverse event
- Any adverse event that, according to clinical judgment of the investigator, is considered as a definite contra-indication to proceeding with the study procedures.
- The use of concomitant, chronic medication active on the immune system (steroids, immunosuppressive agents)
- Pregnancy
- Withdrawal of informed consent by volunteer
- Completely lost to follow-up

6.5 Follow-up of subjects withdrawn from treatment

If a subject fails to appear for a follow-up examination, extensive effort (i.e. documented phone calls and certified mail) will be undertaken to locate or recall him or at least to determine his health status. These efforts will be documented in the subject's CRF and source documents. In the event that a volunteer discontinues the study for any reason, he/she will be required to complete all safety follow-up as appropriate, as determined by the principle investigator and the safety monitor. All volunteers who have been exposed to the bites of infectious mosquitoes will be included in the safety analysis.

6.6 Premature termination of the study

The study may be discontinued for the following reasons:

- On advice of the safety monitor
- On advice of the DSMB
- On advice of the investigators
- On advice of the IRB
- Withdrawal of Informed consent by the volunteer

The safety monitor, DSMB, IRB or investigators may decide to put the study on hold based on adverse events, pending discussion with the IRB/DSMB/safety monitor/investigators. Following discussion, it may be decided to terminate the study. Safety reporting procedures are described in section 7.

7. SAFETY REPORTING

7.1 Section 10 WMO event

In accordance to section 10, subsection 1, of the WMO, the investigator will inform the subjects and the reviewing accredited METC if anything occurs, on the basis of which it appears that the disadvantages of participation may be significantly greater than was foreseen in the research proposal. The study will be suspended pending further review by the accredited METC, except insofar as suspension would jeopardise the subjects' health. The investigator will take care that all subjects are kept informed.

7.2 Adverse and serious adverse events

7.2.1 Adverse events

Adverse events are defined as any undesirable experience occurring to a subject during the study, whether or not considered related to trial. An AE can therefore be any unfavorable and unintended sign (including an abnormal laboratory finding), symptom, or disease (new or exacerbated) temporally associated with the use of a medicinal product or study intervention. AEs may include events that occur as a result of protocol-mandated procedures (i.e. invasive procedures, modification of subject's previous therapeutic regimen). All adverse events reported spontaneously by the subject or observed by the investigator or his staff will be recorded.
Abnormal laboratory findings (e.g., clinical chemistry, haematology, urinalysis) or other abnormal assessments that are judged by the investigator to be clinically significant will be recorded as AEs or SAEs if they meet the definition. The investigator will exercise his or her medical and scientific judgment in deciding whether an abnormal laboratory finding or other abnormal assessment is clinically significant.

If there are any complaints not typical for malaria infection, such as chest pain, the volunteer will be evaluated immediately by a qualified clinician using the appropriate clinical tools (eg. ECG or measurement of cardiac enzymes) according to standard hospital care.

7.2.2 Serious adverse events

A serious adverse event is any untoward medical occurrence or effect that at any dose:

- results in death;
- is life threatening (at the time of the event);
- requires hospitalisation or prolongation of existing inpatients' hospitalisation;
- results in persistent or significant disability or incapacity;
- is a congenital anomaly or birth defect;
- is a new event of the trial likely to affect the safety of the subjects, such as an unexpected outcome of an adverse reaction, lack of efficacy of an IMP used for the treatment of a life threatening disease, major safety finding from a newly completed animal study, etc.

All SAEs will be reported through the web portal ToetsingOnline to the IRB that approved the protocol, within 15 days after the sponsor has first knowledge of the serious adverse reactions.
SAEs that result in death or are life threatening will be reported expedited. The expedited reporting will occur not later than 7 days after the responsible investigator has first knowledge of the adverse reaction. This is for a preliminary report with another 8 days for completion of the report.

7.2.3 Adverse Event Data Collection

Safety assessments will be performed, and recorded by the investigators. All adverse events/reactions (solicited and unsolicited), observed by the investigators or by the subject, will be accurately documented in the case report form by the investigators. For each event/reaction the following details will be recorded:

1. description of the event(s)/reaction(s)
2. date and time of occurrence
3. duration
4. intensity
5. relationship with the intervention
6. action taken, including treatment
7. outcome

In addition, symptoms will be ranked as (1) mild, (2) moderate, or (3) severe, depending on their intensity. All adverse events except fever will be judged for their intensity according to the following scale:

- Mild (grade 1): awareness of symptoms that are easily tolerated and do not interfere with usual daily activity
- Moderate (grade 2): discomfort that interferes with or limits usual daily activity
- Severe (grade 3): disabling, with subsequent inability to perform usual daily activity, resulting in absence or required bed rest

For fever, the following scale will be used:

- Mild (grade 1): 37.5 - 38.0°C
- Moderate (grade 2): > 38.0 to 39.0°C
- Severe (grade 3): > 39.0°C

If an AE changes in frequency or intensity during the specified reporting period, the previous description of the AE will be corrected.

When an AE/SAE occurs, it is the responsibility of the investigators to review all documentation (e.g., hospital progress notes, laboratory, and diagnostics reports) related to the event. The investigators will then record all relevant information regarding an AE/SAE on the CRF or SAE Report Form, respectively.

The investigator will attempt to establish a diagnosis of the event based on signs, symptoms, and/or other clinical information. In such cases, the diagnosis should be documented as the AE/SAE and not the individual signs/symptoms.

7.2.4 Assessment of causality

The investigators are obligated to assess the relationship between study procedures and the occurrence of each AE/SAE. The investigators will use clinical judgment to determine the relationship. Alternative causes, such as natural history of the underlying diseases, concomitant therapy, other risk factors and the temporal relationship of the event to the challenge will be considered and investigated. The relationship of the adverse event with the study procedures will be categorized as:

Probable	An adverse event that follows a reasonable temporal sequence from the challenge procedure and cannot be reasonably explained by the known characteristics of the subject's clinical state.
Possible	An adverse event for which insufficient information exists to indicate a high improbability that the event is related to the study procedure.
Not related	An event for which sufficient information exists to indicate that the etiology is unrelated either because of the temporal sequence of events or because of the subject's clinical state or other therapies

7.3 Follow-up of adverse events

All adverse events will be followed until they have abated, or until a stable situation has been reached. Depending on the event, follow up may require additional tests or medical procedures as indicated, and/or referral to the general physician or a medical specialist.

Investigators will follow-up subjects:
With SAEs or those withdrawn from the study as a result of an AE, until the event has resolved, subsided, stabilized, disappeared, the event is otherwise explained, or the subject is lost to follow-up
With other non-serious AEs, until the subject has completed the study or is lost to follow-up.

Clinically significant laboratory abnormalities will be followed up until they have normalized, or until an alternative explanation, that is not related to the study has been provided. Additional information (including but not limited to laboratory results) relative to the subsequent course of such an abnormality will be made available to the safety monitor.

7.1 Local Safety Monitor and Data Safety Monitoring Board (DSMB)

For this study, a local safety monitor is appointed, who is based in the Leiden University Medical Centre and will be involved in the review of severe and serious adverse events and volunteer safety. He is an experienced clinician qualified to evaluate safety data from clinical studies with malaria infections. He is independent of the investigator team. His main responsibility will be the assessment of the events and recommendation regarding halting further immunizations or challenge. Furthermore, an independent Data Safety Monitoring Board has been appointed.

7.1.1 Review of Safety Data by the Safety Monitor and DSMB

A safety report including a list of all reported adverse events and any safety laboratory values outside the normal ranges will be prepared after each immunization, and on day 228, 239 and 253. These reports will be prepared by the Principal Investigator and sent to the Safety Monitor and all clinical investigators involved. The Safety Monitor will review the safety data within 2 working days and if warranted instruct the site to withdraw or treat individual subjects and/or suspend further study procedures. Before each immunization and before the challenge, the safety data is discussed in a meeting between the study investigator and the Safety Monitor. In addition, safety data after the third immunization and on day 253 will be assessed by the Data Safety Monitoring Board. Responsibilities of the Data Safety Monitoring Board are described in the DSMB Charter (section K5).
All serious adverse events will be reported by the Principal Investigator to the Safety Monitor, the Sponsor and the CCMO within 24 hours. Any highly sensitive troponin T value greater than 0.03 µg/L will be reported to the Safety Monitor within 24 hours. Any laboratory values leading to immediate malaria treatment will be reported to the Safety Monitor within 24 hours.
The advice(s) of the DSMB will be notified upon receipt by the sponsor to the CCMO that approved the protocol. With this notification a statement will be included indicating whether the advice will be followed.

8. STATISTICAL ANALYSIS

All challenged volunteers will be included in the intention-to-treat analysis. Complete protection is defined as negative thick smears till day 21 after challenge infection.

Mean time to thick smear positivity, mean duration of parasitemia and maximum peak height parasitemia between the three groups will be assessed by two-tailed student's t-test (if comparing two groups) or a one-way ANOVA (if comparing more than two groups) or non-parametric equivalents.

Development of parasitaemia will be compared between groups using the statistical model, which will provide the estimated number of infected hepatocytes and the parasite multiplication rate per volunteer (Hermsen 2001). Differences between mean number of infected hepatocytes and parasite multiplication rate will be assessed by two-tailed student's t-test (if comparing two groups) or a one-way ANOVA (if comparing more than two groups) or non-parametric equivalents.

The secondary endpoints will be assessed by comparing mean values between the groups using either a two-tailed student's t-test (if comparing two groups) or a one-way ANOVA (if comparing more than two groups) or non-parametric equivalents, paired if pre-immunization values are compared with post immunization values, unpaired if comparisons are made between groups.

For discrete variables (e.g. the number of positive tick smears in a group, the number of positive assays), the chi-squared test or Fisher's exact test will be used (two-tailed).

All adverse events for each volunteer will be tabulated. Adverse events will be analyzed by calculating the proportion of volunteers in each group who report mild, moderate or severe adverse events. The frequency of signs and symptoms will be compared between groups with the chi-square test.

9. ETHICAL CONSIDERATIONS

9.1 Regulation statement

This study will be conducted in accordance with the latest South Africa revision of the Declaration of Helsinki (section K6b), the Medical Research Involving Human Subjects Act (WMO), the ICH Good Clinical Practice, and local regulatory requirements.
The investigators shall be responsible for obtaining Ethics Committee(s) approval (IRB) of the protocol and any subsequent amendments in compliance with local law before the start of the study.

9.2 Recruitment and consent

As soon as the study is approved by the CCMO, advertisements will be placed on different University campuses in the West of the Netherlands and other public places as well as on the intranet of the institutions. The investigators will be responsible for providing adequate verbal and written information regarding the objectives and procedures of the study, the potential risks involved and the obligations of the volunteers. Volunteers will be informed that they will not gain health benefits from this study. Trainees or other students who might be dependent on the investigators or the study group will not be included in the study. After free discussion with the investigator, the volunteer will be given sufficient time to consider participation. Obtaining informed consent and screening of volunteers will not start until full approval is obtained from the CCMO. On the screening visit written informed consent is obtained from the volunteer. A sample volunteer information letter containing this information and informed consent form can be found in sections E1 and E2.
Volunteers will be informed that they can withdraw their informed consent at any time during the study.

9.3 Benefits and risks assessment, group relatedness

Two major areas of ethical concerns are contained within this proposal, namely the use of blood from humans and the use of human volunteers for controlled human malaria infections. All partners in this proposal are aware of and follow the relevant national and international rules and regulations as they pertain to access to material of human origin and clinical research. International agreements such as the Helsinki declaration and the Convention of the Council of Europe on Human Rights and Biomedicine will be observed and respected.

9.3.1 Ethical aspects concerning the production of *P. falciparum* infected mosquitoes

The human blood used in this study is declassified from screened healthy blood donors from the hospital blood bank and parasites are cultured with serum. Continuous culture of drug sensitive *Plasmodium falciparum* isolate NF54 has been routine over the past 2 decades of the Central Animal Facility of the RUNMC. All strains originate from patient material. All culture material is checked for bacterial contamination, mycoplasma and for blood transmitted diseases (HIV, HBV, HCV, HTLV 1+2).

9.3.2 Ethical aspects concerning the use of human volunteers

Infection of human volunteers with malaria has been carried out over several decades including therapeutic use as treatment for neurosyphilis and later for vaccine evaluation. The ability to carry out this type of work is largely based on the relatively low morbidity and (in more recent times) the lack of mortality seen in these studies. The occurrence of a cardiac event in a volunteer participating in a combined phaseI/IIa malaria vaccine trial in Nijmegen has raised intense discussion on the safety of malaria challenge trials with respect to cardiac events. Based on recommendations of the CCMO and an External Scientific Advisory Committee to the European Malaria Vaccine Development Association, this malaria challenge trial protocol has been adjusted (section 1.5).

The only reliable and convincing way to obtain information on the capacity of potential vaccine strategies to induce protection will be testing in human subjects. Of course, the compelling need for a malaria vaccine need to be balanced to the potential risks and discomforts of the volunteers. Explorative studies of a human immunization model for pre-erythrocytic malaria vaccines is of paramount importance and a potentially powerful tool in the difficult decision making process of bringing vaccine candidates into endemic countries

The study will be undertaken in accordance with good clinical practice, according to the standards defined in the EEC directive 91/507/EEC, and in the Directive on Good Clinical Practice in Clinical Trials (ICH GCP, 75/318/EEC, January 1997) and under the principles of the Declaration of Helsinki (section K6b); ethical permission will be sought from the CCMO the Netherlands.

9.4 Compensation for injury

The sponsor has a liability insurance which is in accordance with article 7, subsection 6 of the WMO, covered by the RUNMC insurance for human research. This is provided by Akkermans van Elten Assurantiën BV, Postbus 181, 6660 AD Elst. Volunteer insurance text is provided in section E4.

9.5 Incentives

Volunteers will receive a maximum of 1700 Euros in compensation fee; travel expenses will not be additionally reimbursed. This amount of money is reasonable and in line with Dutch common practice. In case of unexpected medical complications, there will be state-of-the-art medical treatment at full costs covered by the insurance of Radboud University Nijmegen Medical Center.

10. ADMINISTRATIVE ASPECTS AND PUBLICATION

10.1 Handling and storage of data and documents

All parties agree to adhere to the principles of medical confidentiality in relation to Clinical Study Subjects involved in the Clinical Study. Neither party shall disclose the identity of Clinical Study Subjects to third parties without prior written consent of the Clinical Study Subject.

All serum samples, or other volunteer material will be labelled with the volunteer study identification number. The samples will not be labelled with volunteer names or birth dates. Samples will be stored for 15 years.

10.2 Amendments

Amendments are changes made to the research after a favourable opinion by the accredited METC has been given. All amendments will be notified to the CCMO.

10.3 Annual progress report

The sponsor will submit a summary of the progress of the trial to the CCMO once a year. Information will be provided on the date of inclusion of the first subject, numbers of subjects included and numbers of subjects that have completed the trial, serious adverse events/ serious adverse reactions, other problems, and amendments.

10.4 End of study report

The investigator will notify the CCMO of the end of the study within a period of 90 days. The end of the study is defined as the last volunteer's last visit. In case the study is ended prematurely, the investigator will notify the CCMO, including the reasons for the premature termination.
Within one year after the end of the study, the investigator/sponsor will submit a final study report with the results of the study, including any publications/abstracts of the study, to the CCMO.

10.5 Public disclosure and publication policy

The final report will be prepared by the investigators in collaboration with the Radboud University Nijmegen Medical Center (RUNMC), department of Medical Microbiology (MMB) representative. It will be signed by the coordinating and the principal investigator. The protocol and data derived from the trial are the exclusive property of the RUNMC-MMB.

All rights, title, and interests in any inventions, know-how or other intellectual or industrial property rights which are conceived or reduced to practice by site staff during the course of or as a result of the study are hereby assigned to the RUNMC-MMB.

Any publication or presentation related to the trial must be approved by the RUNMC-MMB representative and principle investigator before submission of the manuscript. After publication of the results of the trial, any participating centre may publish or otherwise use its own data provided that any publication of data from the trial gives recognition to the trial group. Either center must have the opportunity to review the proposed abstract, manuscript or presentation at least 14 days before submission for publication/presentation.

Any information identified as confidential must be deleted prior to submission, it being understood that the results of this trial are not to be considered confidential.

10.6 List of Publication and Authorship

The authorized persons as an author of the publication(s) are those who have contributed to the protocol and/or to the analysis of the data, and whose names are listed on the flyleaf. According to the main topic of the publication, the first author will be the greatest contributing investigator (or biological evaluator).

11. REFERENCES

Abdulla S, Oberholzer R, Juma O, Kubhoja S, Machera F, Membi C, et al. *Safety and immunogenicity of RTS,S/AS02D malaria vaccine in infants*. The New England journal of medicine. 2008 Dec 11;359(24):2533-44.

Accapezzato D, Visco V, Francavilla V, Molette C, Donato T, Paroli M, Mondelli MU, Doria M, Torrisi MR, Barnaba V. *Chloroquine enhances human CD8+ T cell responses against soluble antigens in vivo.* J Exp Med. 2005 Sep 19;202(6):817-28. Epub 2005 Sep 12.

Balog CIA, Meissner A, Goraler S et al. *Metabonomic investigation of human Schistosoma mansoni infection.* Mol. Biosyst. 7(5), 1473-1480 (2011)

Beeson JG, Osier FH, Engwerda CR. *Recent insights into humoral and cellular immune responses against malaria.* Trends in parasitology. 2008 Dec;24(12):578-84.

Belizaire R, Unanue ER. *Targeting proteins to distinct subcellular compartments reveals unique requirements for MHC class I and II presentation.* Proc Natl Acad Sci U S A. 2009 Oct 13;106(41):17463-8.

Belnoue E, Costa FT, Frankenberg T, Vigario AM, Voza T, Leroy N, et al. *Protective T cell immunity against malaria liver stage after vaccination with live sporozoites under chloroquine treatment.* J Immunol. 2004 Feb 15;172(4):2487-95.

Breman JG, Plowe CV. *A malaria vaccine for control: more progress*. The Journal of infectious diseases. 2009 Aug 1;200(3):317-20.

Bygbjerg IC, Theander TG, Andersen BJ, Flachs H, Jepsen S, Larsen PB. *In vitro effect of chloroquine, mefloquine and quinine on human lymphocyte proliferative responses to malaria antigens and other antigens/mitogens.* Trop Med Parasitol. 1986 Sep;37(3):245-7.

Bygbjerg IC, Svenson M, Theander TG, Bendtzen K. *Effect of antimalarial drugs on stimulation and interleukin 2 production of human lymphocytes*. Int J Immunopharmacol. 1987;9(4):513-9.

Chulay JD, Schneider I, Cosgriff TM, Hoffman SL, Ballou WR, Quakyi IA, et al. *Malaria transmitted to humans by mosquitoes infected from cultured Plasmodium falciparum.* The American journal of tropical medicine and hygiene. 1986 Jan;35(1):66-8.

Church LW, Le TP, Bryan JP, Gordon DM, Edelman R, Fries L, et al. *Clinical manifestations of Plasmodium falciparum malaria experimentally induced by mosquito challenge.* The Journal of infectious diseases. 1997 Apr;175(4):915-20.

Coban C, Ishii KJ, Kawai T, Hemmi H, Sato S, Uematsu S, Yamamoto M, Takeuchi O, Itagaki S, Kumar N, Horii T, Akira S. *Toll-like receptor 9 mediates innate immune activation by the malaria pigment hemozoin.* J Exp Med. 2005 Jan 3;201(1):19-25.

Collins WE, Contacos PG. *Immunization of monkeys against Plasmodium cynomolgi by X-irradiated sporozoites.* Nature: New biology. 1972 Apr 12;236(67):176-7.

Doolan DL, Dobano C, Baird JK. *Acquired immunity to malaria*. Clinical microbiology reviews. 2009 Jan;22(1):13-36.

Epstein JE, Giersing B, Mullen G, Moorthy V, Richie TL. *Malaria vaccines: are we getting closer?* Current opinion in molecular therapeutics. 2007 Feb;9(1):12-24.

Epstein JE, Rao S, Williams F, Freilich D, Luke T, Sedegah M, et al. *Safety and clinical outcome of experimental challenge of human volunteers with Plasmodium falciparum-infected mosquitoes: an update. The Journal of infectious diseases.* 2007 Jul 1;196(1):145-54.

Garulli B, Stillitano MG, Barnaba V, Castrucci MR. *Primary CD8+ T-cell response to soluble ovalbumin is improved by chloroquine treatment in vivo.* Clin Vaccine Immunol. 2008 Oct;15(10):1497-504.

Hermsen CC, Telgt DS, Linders EH, van de Locht LA, Eling WM, Mensink EJ, et al. *Detection of Plasmodium falciparum malaria parasites in vivo by real-time quantitative PCR.* Molecular and biochemical parasitology. 2001 Dec;118(2):247-51.

Hermsen CC, de Vlas SJ, van Gemert GJ, Telgt DS, Verhage DF, Sauerwein RW. *Testing vaccines in human experimental malaria: statistical analysis of parasitemia measured by a quantitative real-time polymerase chain reaction.* The American journal of tropical medicine and hygiene. 2004 Aug;71(2):196-201.

Hisaeda H, Tetsutani K, Imai T, Moriya C, Tu L, Hamano S, Duan X, Chou B, Ishida H, Aramaki A, Shen J, Ishii KJ, C, Akira S, Takeda K, Yasutomo K, Torii M, Himeno K. *Malaria parasites require TLR9 signaling for immune evasion by activating regulatory T cells.* J Immunol. 2008 Feb 15;180(4):2496-503.

Hoffman SL, Goh LM, Luke TC, Schneider I, Le TP, Doolan DL, et al. *Protection of humans against malaria by immunization with radiation-attenuated Plasmodium falciparum sporozoites.* The Journal of infectious diseases. 2002 Apr 15;185(8):1155-64.

Hoffman SL, Billingsley PF, James E, Richman A, Loyevsky M, Li T, et al. *Development of a metabolically active, non-replicating sporozoite vaccine to prevent Plasmodium falciparum malaria*. Human vaccines. 2010 Jan 21;6(1).

Kyburz D, Brentano F, Gay S. *Mode of action of hydroxychloroquine in RA-evidence of an inhibitory effect on toll-like receptor signaling.* Nat Clin Pract Rheumatol. 2006 Sep;2(9):458-9.

Labro MT, Babin-Chevaye C. *Effects of amodiaquine, chloroquine, and mefloquine on human polymorphonuclear neutrophil function in vitro.* Antimicrob Agents Chemother. 1988 Aug;32(8):1124-30.

Landelijk coördinatiecentrum reizigersadvisering. Malariaprofylaxe bulletin 2011.

Lindon, J.C. and Nicholson, J. K. *Spectoscopic and statistical techniques for information recovery in metabolomics.* Annu. Rev. Anal. Chem. 2008: 1:45-69

Lobel HO, Miani M, Eng T, Bernard KW, Hightower AW, Campbell CC. *Long-term malaria prophylaxis with weekly mefloquine.* Lancet. 1993 Apr 3;341(8849):848-51.

Nevedomskaya E, Mayboroda OA, Deelder AM. *Cross-platform analysis of longitudinal data in metabolomics.* Molecular Biosystems 2011, DOI: 10.1039/c1mb05280b

Nieman AE, de Mast Q, Roestenberg M, Wiersma J, Pop G, Stalenhoef A, et al. *Cardiac complication after experimental human malaria infection: a case report.* Malaria journal. 2009;8:277.

Pacchiarotta T, Hensbergen PJ, Wuhrer M, van Nieuwkoop C, Nevedomskaya E, Derks RJ, Schoenmaker B, Koeleman CA, van Dissel J, Deelder AM, Mayboroda OA. *Fibrinogen alpha chain O-glycopeptides as possible markers of urinary tract infection.* J Proteomics. 2012 Jan 4;75(3):1067-73. Epub 2011 Oct 31.

Pedersen BK, Bygbjerg IC, Theander TG, Andersen BJ. *Effects of chloroquine, mefloquine and quinine on natural killer cell activity in vitro.* An analysis of the inhibitory mechanism. Allergy. 1986 Sep;41(7):537-42.

Pombo DJ, Lawrence G, Hirunpetcharat C, Rzepczyk C, Bryden M, Cloonan N, et al. *Immunity to malaria after administration of ultra-low doses of red cells infected with Plasmodium falciparum.* Lancet. 2002 Aug 24;360(9333):610-7.

Roestenberg M, McCall M, Hopman J, Wiersma J, Luty AJ, van Gemert GJ, et al. *Protection against a malaria challenge by sporozoite inoculation.* The New England journal of medicine. 2009 Jul 30;361(5):468-77.

Roestenberg M, Teirlinck AC, McCall MB, Teelen K, Makamdop KN, Wiersma J, Arens T, Beckers P, van Gemert G, van de Vegte-Bolmer M, van der Ven AJ, Luty AJ, Hermsen CC, Sauerwein RW. *Long-term protection against malaria after experimental sporozoite inoculation: an open-label follow-up study.* Lancet. 2011 May 21;377(9779):1770-6. Epub 2011 Apr 22.

Sauerwein RW, Bijker EM, Richie TL. *Empowering malaria vaccination by drug administration*. Curr Opin Immunol. 2010 Jun;22(3):367-73.

Schlagenhauf P, Adamcova M, Regep L, Schaerer MT, Rhein HG. *The position of mefloquine as a 21st century malaria chemoprophylaxis.* Malar J. 2010 Dec 9;9:357. Review.

Silvie O, Semblat JP, Franetich JF, Hannoun L, Eling W, Mazier D. *Effects of irradiation on Plasmodium falciparum sporozoite hepatic development: implications for the design of pre-erythrocytic malaria vaccines.* Parasite immunology. 2002 Apr;24(4):221-3.

Singer BH, Utzinger J, Ryff CD, Wang Y, Holmes E. *Chapter 11 - Exploiting the Potential of Metabonomics in Large Population Studies. Three Venues.* In: The Handbook of Metabonomics and Metabolomics, John CL, Jeremy KN, Elaine Holmesa2 - John C. Lindon JKN,Elaine H. Elsevier Science B.V., Amsterdam 289-325 (2007)

Steffen R, Fuchs E, Schildknecht J, Naef U, Funk M, Schlagenhauf P, Phillips-Howard P, Nevill C, Stürchler D. *Mefloquine compared with other malaria chemoprophylactic regimens in tourists visiting east Africa.* Lancet. 1993 May 22;341(8856):1299-303.

Suhre K, Shin SY, Petersen AK, Mohney RP, Meredith D, Wägele B, Altmaier E; CARDIoGRAM, Deloukas P, Erdmann J, Grundberg E, Hammond CJ, de Angelis MH, Kastenmüller G, Köttgen A, Kronenberg F, Mangino M, Meisinger C, Meitinger T, Mewes HW, Milburn MV, Prehn C, Raffler J, Ried JS, Römisch-Margl W, Samani NJ, Small KS, Wichmann HE, Zhai G, Illig T, Spector TD, Adamski J, Soranzo N, Gieger C. *Human metabolic individuality in biomedical and pharmaceutical research.* Nature. 2011 Aug 31;477(7362):54-60. doi: 10.1038/nature10354.

Targett GA, Greenwood BM. *Malaria vaccines and their potential role in the elimination of malaria*. Malaria journal. 2008;7 Suppl 1:S10.

Verhage DF, Telgt DS, Bousema JT, Hermsen CC, van Gemert GJ, van der Meer JW, et al. *Clinical outcome of experimental human malaria induced by Plasmodium falciparum-infected mosquitoes.* The Netherlands journal of medicine. 2005 Feb;63(2):52-8.

Walther M, Jeffries D, Finney OC, Njie M, Ebonyi A, Deininger S, Lawrence E, Ngwa-Amambua A, Jayasooriya S, Cheeseman IH, Gomez-Escobar N, Okebe J, Conway DJ, Riley EM. *Distinct roles for FOXP3 and FOXP3 CD4*

T cells in regulating cellular immunity to uncomplicated and severe Plasmodium falciparum malaria. PLoS Pathog. 2009 Apr;5(4):e1000364. Epub 2009 Apr 3.

Wang TJ, Larson MG, Vasan RS, Cheng S, Rhee EP, McCabe E, Lewis GD, Fox CS, Jacques PF, Fernandez C, O'Donnell CJ, Carr SA, Mootha VK, Florez JC, Souza A, Melander O, Clish CB, Gerszten RE. *Metabolite profiles and the risk of developing diabetes.* Nat Med. 2011 Apr;17(4):448-53. Epub 2011 Mar 20.
